# Effectiveness of a Mental Health Service Navigation Website (Link) for Young Adults: Randomized Controlled Trial

**DOI:** 10.2196/13189

**Published:** 2019-10-17

**Authors:** Lena Sanci, Sylvia Kauer, Sharmala Thuraisingam, Sandra Davidson, Ann-Maree Duncan, Patty Chondros, Cathrine Mihalopoulos, Kerrie Buhagiar

**Affiliations:** 1 Department of General Practice University of Melbourne Parkville Australia; 2 Deakin Health Economics, Institute for Health Transformation School of Health and Social Development Deakin University Burwood Australia; 3 ReachOut Australia Sydney Australia

**Keywords:** adolescent, young adult, internet, web archives as topic, mental health, mental disorders, help-seeking behavior, mental health services, affect

## Abstract

**Background:**

Mental health and substance use disorders are the main causes of disability among adolescents and young adults yet fewer than half experiencing these problems seek professional help. Young people frequently search the Web for health information and services, suggesting that Web-based modalities might promote help-seeking among young people who need it. To support young people in their help-seeking, we developed a Web-based mental health service navigation website called *Link. Link* is based on the Theory of Planned Behavior and connects young people with treatment based on the type and severity of mental health symptoms that they report.

**Objective:**

The study aimed to investigate the effect of Link on young people’s positive affect (PA) compared with usual help-seeking strategies immediately post intervention. Secondary objectives included testing the effect of Link on negative affect (NA), psychological distress, barriers to help-seeking, and help-seeking intentions.

**Methods:**

Young people, aged between 18 and 25 years, were recruited on the Web from an open access website to participate in a randomized controlled trial. Participants were stratified by gender and psychological distress into either the intervention arm (Link) or the control arm (usual help-seeking strategies). Baseline, immediate postintervention, 1-month, and 3-month surveys were self-reported and administered on the Web. Measures included the PA and NA scales, Kessler psychological distress scale (K10), barriers to adolescent help-seeking scale (BASH), and the general help-seeking questionnaire (GHSQ).

**Results:**

In total 413 young people were recruited to the trial (intervention, n=205; control, n=208) and 78% (160/205) of those randomized to the intervention arm visited the *Link* website. There was no evidence to support a difference between the intervention and control arms on the primary outcome, with PA increasing equally by approximately 30% between baseline and 3 months in both arms. NA decreased for the intervention arm compared with the control arm with a difference of 1.4 (95% CI 0.2-2.5) points immediately after the intervention and 2.6 (95% CI 1.1-4.1) at 1 month. K10 scores were unchanged and remained high in both arms. No changes were found on the BASH or GHSQ; however, participants in the intervention arm appeared more satisfied with their help-seeking process and outcomes at 1 and 3 months postintervention.

**Conclusions:**

The process of prompting young people to seek mental health information and services appears to improve their affective state and increase help-seeking intentions, regardless of whether they use a Web-based dedicated youth-focused tool, such as *Link*, or their usual search strategies. However, young people report greater satisfaction using tools designed specifically for them, which may encourage future help-seeking. The ability of Web-based tools to match mental health needs with appropriate care should be explored further.

**Clinical Trial:**

Australian New Zealand Clinical Trials Registry ACTRN12614001223628; https://www.anzctr.org.au/Trial/Registration/TrialReview.aspx?id=366731

## Introduction

### Background

Mental ill health is a leading health burden affecting 1 in 4 young people worldwide [1-3] with detrimental effects on relationships, academic achievement, work life, and general well-being [4], which often continue into adulthood [5]. Following investment in mental health reforms in Australia to provide accessible mental health services to young people [6], the rates of help-seeking for mental health problems appear to be improving; however, between 35% and 65% of those in need still fail to seek professional help [7].

Barriers to help-seeking for mental health problems among young people are well documented [8]. Young people may not recognize their symptoms as a mental health problem or know that effective treatments exist [9,10]. Some young people may not be ready to seek help [4] or their symptoms may go undetected by health professionals [11]. The stigma associated with mental illness may also prevent young people from seeking help [12]. Not knowing where or how to access services, perceived costs and inconvenience in accessing care, and fears of being judged or of breaches in confidentiality are other important barriers perceived by young people [10]. Interventions that reduce these barriers and provide a positive experience of help-seeking are needed, particularly interventions that facilitate access to treatments [13] and those that aim to increase young people’s readiness to seek help [14].

Most young people have lived their entire lives in a digital media–saturated world and are highly likely to use the internet to search for health information [15-18]. This suggests that electronic health interventions have the potential to facilitate help-seeking among young people. However, a recent systematic review of Web-based interventions to increase mental health help-seeking revealed poor quality studies and little evidence of impact on actual help-seeking behavior or on the likely precursors of help-seeking, such as mood, perceived barriers, and intentions to seek help [16]. Furthermore, internet interventions often lack or neglect to outline a theoretical foundation, limiting understanding about which elements of a program may be beneficial and why [16].

In response to the deficiencies identified in the literature, we developed *Link*, a website designed to assist young people to find accessible Web-based and computer-based mental health services appropriate for their mental health needs. *Link* was developed in accordance with the Medical Research Council guidelines for complex interventions [19,20]. We undertook a comparative review of relevant behavior change and help-seeking theories and selected the Theory of Planned Behavior on which to base our program logic and ultimately the functional elements of the technology design; a description of this process has been previously published [21]. In the development phase, participatory design [22] with young people was used to understand the features important to include in *Link* that would facilitate youth engagement [23]. The program logic of *Link* thus proposes that by improving attitudes, beliefs, and perceived control of help-seeking, and reducing barriers toward help-seeking, positive affect (PA), and intentions to seek help will increase, which in turn will increase actual help-seeking behaviors [24].

We conducted a pilot randomized controlled trial (RCT) of *Link* with 51 (intervention, n=24; control, n=27) young people aged between 18 and 25 years [25]. Results indicated that *Link* was well accepted by young people and that a larger RCT investigating the effectiveness of *Link* was feasible with minor refinements, including simplifying the sign-up process and removing a link to Google for the control participants. The pilot study also revealed, in keeping with other studies, that help-seeking intentions and behaviors were difficult to define, with no current psychometrically sound measures routinely used in previous studies [26], and that a primary outcome measure other than help-seeking intentions and behaviors should be used in the RCT. On the basis of young people’s typical barriers to seeking help, theoretical considerations, and the availability of a well-validated measure, we chose PA immediately after using a help-seeking strategy as the primary outcome. The theoretical considerations were based on the construct of PA and its potential role as an intermediary in help-seeking. PA reflects the degree to which a person feels alert, active, and enthusiastic [27]. High PA is characterized by energy, concentration, and engagement. It was hypothesized that young people concerned about their mental health and facing barriers to seeking help, such as knowing where to go, what to expect from each service, and perceived stigma and isolation [28], would experience rapid relief of distress when engaging with the features of *Link,* such as immediate return of tailored options for seeking help, personal stories from others with the same symptoms, practical self-care tips, and preparation for what to expect when accessing a service. PA was thus hypothesized to promote engagement with the help-seeking process. This potential intermediary role of PA for help-seeking intentions and behaviors can also be explained by its association with increased coping strategies, positive meaning of issues, connections with others, self-esteem, and validation from others [29].

### Objectives

The primary objective of this study was to assess the impact of *Link* on young people’s PA compared with usual help-seeking strategies immediately post intervention. Secondary objectives were to compare the intervention and control participants on measures of PA 1 and 3 months after using *Link* and on negative affect (NA), psychological distress, barriers to seeking help, and help-seeking intentions at 1 and 3 months postintervention.

## Methods

### Study Design

This was an Australian-based individually randomized controlled trial conducted between November 27, 2014 (first participant recruited) and July 4, 2015 (last follow-up survey completed). All study procedures were conducted on the Web. People aged between 18 and 25 years were randomly allocated in a ratio of 1:1 to either the *Link* (intervention) arm or the usual help-seeking strategy (control) arm. Both arms were followed for 3 months. A secure server at the University of Newcastle, Australia (QuON) was used to manage the trial phases and to collect and store deidentified survey responses. The study was approved by the University of Melbourne Human Research Ethics Committee (ID.1341063.4).

### Participants

#### Eligibility Criteria

Participants were eligible if they were aged between 18 and 25 years, living in Australia, and had sufficient English and computer literacy to complete the survey measures and navigate the *Link* website.

#### Recruitment

A digital marketing company (Profero) was responsible for participant recruitment. The marketing strategy was in English and comprised electronic direct mail, social media, Web-based advertising (eg, Facebook, Gumtree, and Google), and snowballing. Web-based advertisements were directed at young adults between the ages of 18 and 25 years who lived in Australia (Facebook; Multimedia Appendix 1), anyone who searched for mental health information and lived in Australia (Google; Multimedia Appendix 1), and under the community advertisements on Gumtree. Those who clicked on the link to the study in the advertisement were assessed for eligibility using a Web-based form and young people fulfilling the inclusion criteria were invited to participate in the study. Short message service (SMS) text messaging and email reminders were sent to the participants 6 to 10 days after a positive eligibility assessment (for baseline survey), the randomization date (for postintervention survey), the due date for the 1-month survey, and the due date for the 3-month survey. Participants were reimbursed with an Aus $15 gift card for completing each of the first 2 surveys and an Aus $20 gift card for completing the final survey.

### Intervention

*Link* is a self-directed mental health help-seeking service navigation website designed to guide young people to appropriate Web-based and computer-based sources of mental health information and care. It was designed by the research team in conjunction with young people and developed by the software company Tigerspike. The theoretical basis and rationale for each feature of *Link* has been published previously [23,24].

Multimedia Appendix 2 shows screenshots from a computer or tablet of how users move through the program. On the landing page (slide 1), clicking on *get started* guides users through a 3-step self-assessment process. First, the user is asked to select from a list of symptoms, expressed in language co-designed by young people, the one that best reflects how they are feeling (slide 2). The symptoms map to 8 domains: anxiety and depression; bullying; alcohol and drug problems; issues with eating, weight, and body image; relationship difficulties; suicidal intent; and self-harm. Second, users are asked to rate the degree to which the symptoms are affecting them using an interactive pictorial 5-point sliding scale ranging from 1=*I’m OK* to 5=*It’s a huge deal* (slide 3). Third, they select their service preference, that is, face-to-face, phone helpline, Web-based chat or email therapy, or Web-based information and self-help (slide 4). On the basis of information provided in steps 1 to 3, *Link* presents 3 service recommendations from a directory of 31 youth-friendly services (slide 5). Users can click for more service options if required. Information is provided for all services including what to expect when using the service, how the service works, the cost of the service (if any), and a link to the service’s website or location (slide 6). The program also recommends a suitable service modality based upon the severity of the issue. For example, if the user selected *online information* as a service preference for severe thoughts of self-harm, *Link* would also suggest a 24-hour telephone helpline. An emergency contact button also appears at the top of each page for users experiencing high levels of distress (slide 7).

To accommodate those young people who are unable to explain exactly what is bothering them, a list of symptoms is displayed (slide 8). Clicking on the most fitting possibility produces a number of options that map to the 8 domains described above (slide 9). Once the user confirms their main issue, they will re-enter the *Link* program (at slide 3).

Mental health facts and peer-stories were also embedded in *Link*. These features were designed to engage users, influence subjective norms around help-seeking, and increase mental health literacy. Users could access *Link* using either a computer, tablet, or mobile phone. These different platforms were all considered in the design of *Link*, with the mobile display also shown (slide 10). Intervention participants could use *Link* as often as they wished throughout the study, up to the time they completed their last follow-up measure, or they were more than 3 weeks past the due date for their 3-month follow-up survey. This provided them with the opportunity to explore multiple problems that they may have experienced during the study period.

### Study Protocol

Young people who met the eligibility criteria and provided informed consent registered for the trial using their email address and a self-generated password. Email addresses and passwords allowed all participants to login and complete surveys at each wave and use the *Link* program (intervention arm only). Immediately following registration, all participants completed the baseline survey, after which they were randomly allocated to receive *Link* (intervention arm) or be directed to a page with the following instructions (control arm):

We want to know what you normally do to seek help. Please search for information and support for an issue or problem you are currently facing using strategies you normally use to seek help

Individuals who partially completed the baseline survey or did not complete the randomization process were sent email and SMS reminders 4, 7, and 14 days after beginning the enrollment process. Individuals who completed the enrollment process in less than 28 days were considered enrolled in the study.

Immediately after randomization, participants were provided with a link to the postintervention survey to be completed immediately after using the *Link* program (intervention arm) or they undertook usual help-seeking strategies (control arm), which may have been the same day as they were randomized. Participants were sent reminders via email and SMS with the link to the survey 1, 4, and 7 days later. Participants who did not complete the postintervention assessments within 14 days from completing baseline were considered nonresponders.

A month after completing the baseline survey, participants received an email and SMS providing a link to the 1-month follow-up survey, with reminders sent 1 and 2 weeks later. Participants who did not complete this survey after 3 weeks were considered nonresponders. After 3 months from baseline, participants received an email and SMS to complete the 3-month follow-up survey. Reminders were sent 1 and 2 weeks later. Participants who did not complete the 3-month follow-up survey within 3 weeks from the first reminder were treated as nonresponders.

Checks for valid input data were programmed into QuON, so that only valid survey responses could be entered. Some questions had to be answered before continuing to the next page. All activity in *Link* was tracked, recorded, and linked to the intervention participants’ unique identification number. The study design also included an economic evaluation which is described in full in a companion paper [30].

#### Measurement Time Lines

All outcome measures were collected from both arms at baseline, 1 month, and 3 months postintervention. PA and NA and satisfaction were also measured 2 weeks (immediately) after randomization in both arms to capture effects after first using their respective allocated intervention.

### Primary Outcome Measure

#### Positive Affect

PA was measured using the PA scale of the positive and negative affect scale (PANAS) [27]. A PA score was calculated by adding the 10 PA items. The PA score can range between 10 and 50, with higher scores representing higher level of PA. The 10-item PA scale has high internal consistency, is valid and reliable over a 2-month period, and is sensitive to mood fluctuations if used with short-term instructions (eg, now) or to stable traits if used with longer-term instructions (eg, past year) [27].

### Secondary Outcome Measures

Secondary outcomes included PA at all other follow-up points, and the other measures described below.

#### Negative Affect

NA was measured using the 10-item NA scale of the PANAS [27]. NA reflects an individual’s degree of subjective distress arising from mood states, such as anger, guilt, fear, and nervousness. Low NA is characterized by a state of calmness and serenity. NA is related to self-reported stress, poor coping, and frequency of negative events. Developed alongside the PA scale, the NA scale is also highly internally consistent, largely uncorrelated, and stable at appropriate levels over a 2-month time period (Cronbach alpha reliabilities for intercorrelations and internal consistency reliabilities range from .86 to .90 for PA and from .84 to .87 for NA, with reliabilities unaffected by the time instructions used) [27]. The NA scale was used to indicate if there was an immediate benefit of using *Link* and if any harms arose from either arm. The 10 NA items were added to calculate a total NA score ranging between 10 and 50, with lower scores representing lower levels of NA.

#### Psychological Distress

Psychological distress was measured using the Kessler psychological distress scale (K10) [31]. The K10 has good precision in the 90th to 99th percentile range of the population distribution (standard errors of standardized scores in the range 0.20 to 0.25) and maintains consistent psychometric properties across major sociodemographic subsamples [31]. The K10 strongly discriminates between community cases and noncases of structured clinical interview for diagnostic and statistical manual of mental disorders (IV) [31]. The K10 comprises 10 questions asking about the frequency of depressive and anxiety symptoms in the past 4 weeks. Each item is rated on a 5-point scale (1=none of the time and 5=all of the time) and scores are summed to a possible range of 10 to 50, with higher scores indicating higher distress. For the random allocation, participants with a K10 score less than 20 at baseline were classified as likely to be well, whereas participants scoring 20 or more were classified as likely to have a mental disorder. The K10 is a reliable measure with all items of relevance to young people [32].

#### Barriers to Help-Seeking

Barriers to seeking help for mental health problems were measured using the barriers to adolescents seeking help (BASH) scale [9], adapted by Wilson [33]. This is an 11-item scale that includes questions around knowledge of available resources, mental health stigma, and attitudes to help-seeking. Each item is scored on a 6-point Likert scale (1=strongly agree and 6=strongly disagree); items are reverse scored and added so that higher mean scores indicate increased barriers to help-seeking. The BASH scale has good test-retest reliability, internal reliability, and validity among adolescents [9]; however, it showed no variance in our pilot study and so was not chosen as the primary outcome [25].

#### General Help-Seeking Questionnaire

The general help seeking questionnaire (GHSQ) [33] includes 12 items asking how likely the individual is to seek help for an emotional or personal problem from different services and people. When tested in a population of high school students, the GHSQ was found to have satisfactory reliability and validity and was considered a suitable measure of help-seeking intentions when applied to a range of contexts [33]. Sources of help were classified as informal (eg, intimate partner, friend, parent, and relative/family member); formal (eg, mental health professional, doctor/general practitioner, minister/religious leader, hospital, and medication prescriber); Web-based/phone (phone helpline and Web-based tools/apps); and none (do not seek help from anyone). Each statement was answered on a 7-point Likert scale from 1 (extremely unlikely) to 7 (extremely likely). All items in the formal (4 items), informal (5 items), and Web-based/phone categories (2 items) were averaged for a total score. This measure also showed little variance in the pilot study, which, along with being in the review of measures not psychometrically robust, was why it was not a primary outcome this study [25,26].

#### Satisfaction With *Link*

Participants were also asked 10 items adapted from Retolaza [34] about whether their expectations were met in the postintervention and 1-month and 3-month follow-up surveys [25]. Each item was scored on a 5-point Likert scale (strongly disagree, disagree, neither agree or disagree, agree, and strongly agree).

#### Baseline Characteristics

Demographic information included age, gender, education and employment status, and language spoken at home. Participants were also asked to rate their perception of their mental health at baseline using a 5-point scale from *perfect no illness or problems* to *severe illness* [35]. Participants were asked about their health service use in the past 6 months and whether they had searched the Web for mental health information or services in the previous 2 weeks.

#### Help-Seeking Strategy After Randomization

Both arms were asked about the method they used to seek help in the first 2 weeks (immediate), 1 month, and 3 months after randomization mainly to gain an understanding of methods used by the control arm.

### Sample Size

A sample size estimate of 214 participants (107 per arm) was based on the PA scale of the PANAS with a minimal clinically significant difference in mean scores between the two arms of 2.7, assuming a standard deviation of 7.9, 80% power, and 5% significance level [27]. To test our primary hypothesis that participants in the intervention arm would, on average, report an increase in PA immediately after using *Link* compared with participants in the control arm, we based our sample size calculations on a 1-tailed independent *t* test. Owing to the high attrition rates commonly observed in Web-based recruitment [36-38], the sample size was inflated by two-thirds to 336 young adults (168 participants in each arm).

### Randomization and Masking

A 32-character unique identification code comprising letters and numbers was assigned to each participant. After completing the baseline measures, participants were randomly allocated to either the intervention or control arm using a random allocation sequence generated internally by the QuON computer software (University of Newcastle). Randomization was stratified by gender (male, female) and psychological distress (K10 score <20 and K10 score ≥20; K10 was completed as a baseline measure) using random sequences of block sizes of 4, 6, or 8 within each stratum and an allocation ratio of 1:1. A statistician not involved with the research oversaw this procedure to ensure accuracy and blinding of the research team. Researchers and statisticians involved in the data analysis were blind to the allocation of participants until after data analysis was completed. It was not possible to blind participants to the study arm to which they were assigned as the study information stated that they would be asked to look for services either through usual methods or a Web-based program.

### Data Monitoring and Use

Regular monitoring of survey data on the QuON database and tracking data on the *Link* website was conducted by the researchers by reviewing the tracking logs to ensure that data were being recorded.

### Statistical Analysis

Stata version 13.1 [39] was used for all analyses that used an intention-to-treat approach [40]. Descriptive statistics were used to compare participant baseline characteristics, baseline outcome measures, and health service use between the study arms. Help-seeking strategies used postrandomization and responses to the satisfaction with search strategies (dichotomized according to whether they responded as *Strongly agree/Agree* or *not*) were summarized using counts and percentages by study arm for each follow-up time.

Linear mixed-effects models with random intercepts were used to estimate differences in mean outcome between the study arms at each time point using restricted maximum likelihood estimation. Individual participant data were treated as random effects and an unstructured correlation structure was used to account for the repeated measures. All regression analyses (except K10 score) included randomization stratification factors of gender (male and female) and baseline K10, dichotomized as high (K10 >20) and low/moderate (K10 ≤20) probability of mental disorder and time of follow-up (baseline, immediate, 3 months, and 6 months) as fixed effects. An interaction term between the study arm and follow-up time was also included, except at baseline where the study arm means were constrained to be equal.

We were unable to fit a linear mixed-effects regression model for the PA and NA scores at 2 weeks (immediate) as they were correlated with their respective scores at baseline. Thus, for these 2 outcomes at 2 weeks, we used linear regression to estimate the mean differences in the outcome between study arms, with adjustments for gender and baseline K10 dichotomized score. In a secondary analysis, estimates for all outcomes were also adjusted by whether participants had searched the Web for mental health services in the 2 weeks (yes/no) before commencement of the study. Goodness of fit of the models were assessed using residual plots.

Under the fitted linear mixed-effects model, missing data were assumed to be missing at random. A sensitivity analysis was performed using a pattern-mixture model to assess the robustness of this assumption for the PANAS (details provided in Multimedia Appendix 3).

## Results

### Overview

**Figure 1:**
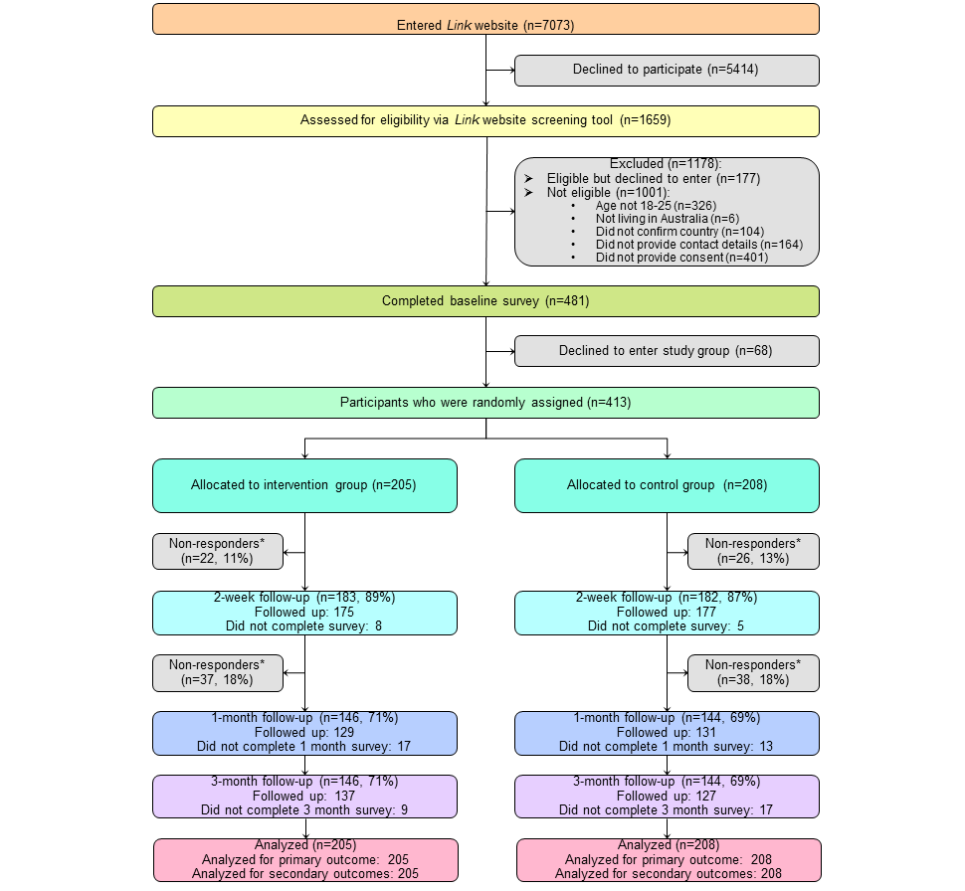
Trial flow diagram. Nonresponders were participants who did not complete the remaining surveys (note: the denominator used for the percentages was the number of participants allocated to the intervention arm (n=205) and the control arm (n=208), respectively).

Participant flow through the study is shown in Figure 1. The *Link* study website was visited by 7073 people. Of these, 658 (653/7073, 9.3%) met the eligibility criteria and provided consent. Of those consenting, 481 (481/653, 73%) participants completed the baseline survey with 68 (68/481,14.1%) discontinuing at this point, leaving 413 (413/481, 85.9%) participants for randomization. Attrition rates over time were similar between the 2 arms. Characteristics between young people who withdrew and those who completed the study were similar, with the mean age of participants at baseline being 20.7 (SD 2.3) and 21.3 (SD 2.1) in the intervention and control arms, respectively (0 missing responses in both arms). Results are presented in Multimedia Appendix 4.

Baseline characteristics of participants are summarized in Table 1. The mean age of participants was 20.1 (SD 2.3) and 21.3 (SD 2.4) in the intervention and control arms, respectively (0 missing responses in both arms). Over 80% of the participants were female and 14% from non-English speaking backgrounds. In total, 37% (148/405) of participants reported moderate-to-severe mental health ratings and 68% (278/411) reported 2 or more psychological issues. Baseline characteristics were similar in both arms. Health service use was also similar (Table 2) except that a larger proportion of intervention participants (38.5%) had searched the Web for mental health services in the previous 2 weeks compared with control participants (26.0%).

### Outcomes

#### Primary Outcome

**Figure 2:**
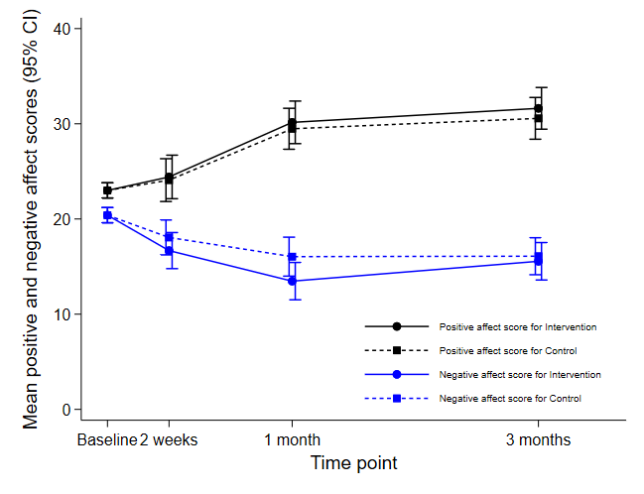
Estimated mean positive and negative affect scores at baseline, immediately postintervention, 1 month, and 3 months for intervention and control arms.

There was no evidence to support a difference between arms for mean PA at any of the follow-up time points postintervention (Table 3). However, Figure 2 shows that compared with mean baseline PA score, both arms showed approximately 30% improvement at 3 months’ follow-up.

**Table 1:** Participant characteristics by intervention and control arms at baseline (percentages may not sum to 100% because of rounding and totals may vary because of missing responses; counts and percentages presented unless otherwise stated).

Participant characteristic			*Link* (n=205)		Control (n=208)	
			Value, n (%)	Value of missing responses^a^, n (%)	Value, n (%)	Value of missing responses^a^, n (%)
Female			171 (83.4)	0 (0)	173 (83.2)	0 (0)
Neither working nor studying			14 (7.9)	28 (13.7)	16 (9.0)	28 (13.5)
Socioeconomic advantage^b^			92 (44.9)	65 (31.7)	102 (49.0)	68 (32.7)
Rural^a^			52 (37.1)	65 (31.7)	43 (30.5)	67 (32.2)
English not spoken at home			32 (15.6)	0 (0)	25 (12.0)	0 (0)
Aboriginal or Torres Strait Islander			4 (2.0)	4 (2.0)	6 (3.0)	5 (2.4)
**Highest education level**				1 (0.5)		0 (0)
	Did not complete secondary school		10 (4.9)	—	14 (6.8)	—
	Completed/partially completed years 11/12		104 (51.0)	—^c^	99 (47.6)	—
	Certificate or diploma		47 (23.0)	—	48 (23.1)	—
	Undergraduate degree		37 (18.1)	—	41 (19.7)	—
	Post graduate degree, masters, or PhD		6 (2.9)	—	6 (2.9)	—
**Mental health rating (K10)**				6 (2.9)		2 (1.0)
	No illness/problems		21 (10.6)	—	26 (12.6)	—
	Some symptoms but no disease		68 (34.2)	—	60 (29.1)	—
	Minor illness		34 (17.1)	—	48 (23.3)	—
	Moderate illness		61 (30.7)	—	57 (27.7)	—
	Severe illness		15 (7.5)	—	15 (7.3)	—
**Self-reported issues^d^**			—	1 (0.5)	—	1 (0.5)
	**Number of issues reported**					
		None	22 (10.8)	—	16 (6.3)	—
		One	40 (19.6)	—	58 (28.0)	—
		Two or more	142 (69.6)	—	136 (65.8)	—
	**Issue reported by participants^d^**					
		Often stressed, worried, or down	162 (79.4)	—	165 (79.7)	—
		Often stressing about body, food, or exercise	104 (51.0)	—	123 (59.4)	—
		Worried about my drug or alcohol use	16 (7.8)	—	12 (5.8)	—
		Harming myself	9 (4.4)	—	16 (7.7)	—
		Thinking about ending my life	23 (11.3)	—	26 (12.6)	—
		Being bullied on the Web, school, or work	4 (2.0)	—	5 (2.4)	—
		Having problems with people close to me	66 (32.4)	—	57 (27.5)	—
**Primary and secondary outcomes**				0 (0)		0 (0)
	Positive affect, mean (SD)		22.9 (8.3)	—	23.1 (7.8)	—
	Negative affect, mean (SD)		20.1 (7.8)	—	20.8 (9.3)	—
	Psychological distress (K10), mean (SD)		27.9 (9.2)	—	27.7 (9.5)	—
	Barriers to Seeking Help, mean (SD)		37.6 (9.6)	—	37.7 (9.0)	—

^a^Number of missing responses presented as count and percentage of total allocated to the intervention arm (n=205) and control arm (n=208), respectively.

^b^Index of Relative Socio-Economic Advantage and Disadvantage Australian Bureau of Statistics.

^c^Not applicable.

^d^Subcategories are not mutually exclusive.

**Table 2:** Health service use by the intervention and control arms at baseline (percentages may not sum to 100% because of rounding).

Health service use		*Link* (n=205), n (%)	Control (n=208), n (%)
**Health services type/treatments last 6 months** ^a^			
	GP^b^	135 (65.9)	128 (61.5)
	Psychologist	47 (22.9)	56 (26.9)
	Psychiatrist	18 (8.8)	27 (13.0)
	Headspace service (GP, psychologist, counsellor)	23 (11.2)	22 (10.6)
	Other	16 (7.8)	12 (5.8)
**Use of health services/treatments in last 6 months combinations (mutually exclusive)**			
	Not using any services	59 (28.8)	60 (28.9)
	GP only	72 (35.1)	68 (32.7)
	GP and psychologist	23 (11.2)	29 (13.9)
	GP and psychiatrist	3 (1.5)	4 (1.9)
	GP and headspace	10 (4.9)	1 (1.0)
	GP and 1 other service	6 (2.9)	4 (1.9)
	GP and 2 services	14 (6.8)	14 (6.7)
	GP and 3 or more services	7 (3.4)	7 (3.4)
	1 service (not GP)	11 (5.4)	13 (6.3)
	2 or more services (not GP)	0 (0)	7 (3.4)
Web-based mental health search, last 2 weeks		79 (38.5)	54 (26.0)

^a^Subcategories not mutually exclusive.

^b^GP: general practitioner.

**Table 3:** Estimated means and between-arm differences on primary and secondary outcomes at each follow-up time (N=413). Estimated using linear mixed-effects regression, except for outcomes PA and NA immediately postintervention that were estimated using linear regression. All models were adjusted by gender, K10, and whether participants had searched for Web-based mental health services in the last 2 weeks. Estimates using model with no adjustment for Web-based health services search were similar (not shown).

Outcome		*Link,* n=205		Control, n=208		Difference	95% CI	*P* value
		Mean	95% CI	Mean	95% CI			
**Positive affect**								
	Baseline^a^	23.0	22.2 to 23.8	23.0	22.2 to 23.8	—^b^	—	—
	Immediate	24.4	22.1 to 26.7	24.1	21.8 to 26.3	0.3	−1.1 to 1.8	.65
	1 month	30.2	27.9 to 32.4	29.5	27.3 to 31.6	0.7	−1.0 to 2.4	.44
	3 months	31.6	29.4 to 33.8	30.6	28.4 to 32.8	1.0	−0.7 to 2.8	.24
**Negative affect**								
	Baseline^a^	20.4	19.6 to 21.3	20.4	19.6 to 21.3	—	—	—
	Immediate	16.7	14.8 to 18.6	18.1	16.2 to 19.9	−1.4	−2.5 to −0.2	.02
	1 month	13.5	11.5 to 15.4	16.0	14.0 to 18.1	−2.6	−4.1 to −1.1	.001
	3 months	15.6	13.6 to 17.5	16.1	14.1 to 18.0	−0.5	−2.0 to 1.0	.49
**Psychological distress (K10)**								
	Baseline^a^	26.2	24.1 to 28.3	26.2	24.1 to 28.3	—	—	—
	1 month	24.2	22.0 to 26.5	25.2	22.9 to 27.4	−0.9	−2.4 to 0.6	.23
	3 months	23.4	21.1 to 25.6	24.4	22.1 to 26.6	−1.0	−2.5 to 0.6	.21
**Barriers to seeking help**								
	Baseline^a^	34.9	32.7 to 37.2	34.9	32.7 to 37.2	—	—	—
	1 month	35.4	33.0 to 37.9	35.0	32.6 to 37.5	0.4	−1.3 to 2.0	.65
	3 months	33.8	31.4 to 36.3	34.6	32.1 to 37.0	−0.8	−2.5 to 1.0	.41

^a^Estimated mean (95% CI) of baseline outcome for the two study arms are the same because they were constrained to be equal in the mixed-effects model.

^b^Not applicable.

#### Secondary Outcomes

There was evidence to support a reduction in the mean NA for the intervention arm compared with the control arm at the immediate and 1-month follow-up time points (Table 3). However, the intervention effect diminished at 3-month follow-up. Sensitivity analyses for PA and NA scores showed that the findings are unlikely to change when the departures from missing at random assumption are assumed to occur in the same way in both study arms. Study conclusions could change if departures from the missing at random assumption differed in the 2 study arms, but we considered this an unlikely scenario as the participants with missing data were similar in the 2 study arms (see Multimedia Appendix 3 for details).

Mean scores on the K10 and the BASH remained relatively stable for the duration of the study for both arms and there was no evidence to support between-arm differences at any time point. There was, however, weak evidence to support that there was greater intention to seek general help at 3-month in the intervention arm compared with the control arm (Difference −0.22, 95% CI −0.44 to −0.009; Table 4). There was no difference between *Link* and the young people in the control group in how likely they were to *not seek help from anyone*; however, at 1 and 3 months, they were, on average, less likely to *not seek help from anybody* compared with the baseline responses.

**Table 4:** Estimated means and between-arm differences and respective 95% CI on the general help seeking questionnaire at baseline, 1 month, and 3 months (N=413; estimated using linear mixed-effects regression adjusted for gender, K10, and whether participants had searched for Web-based mental health services in the last 2 weeks).

Help-seeking behavior		*Link,* (n=205)		Control, (n=208)		Difference	95% CI	*P* value
		Mean	95% CI	Mean	95% CI			
**Total score**								
	Baseline^a^	3.7	3.4 to 3.9	3.7	3.4 to 3.9	—^b^	—	—
	1 month	3.4	3.1 to 3.7	3.4	3.2 to 3.7	0.046	−0.17 to 0.26	.67
	3 months	3.6	3.4 to 3.9	3.4	3.1 to 3.7	−0.22	−0.44 to −0.009	.04
**Informal**								
	Baselinea^a^	4.1	3.8 to 4**.**4	4.1	3.8 to 4**.**4	—	—	—
	1 month	4	3.6 to 4.4	4.1	3.8 to 4.5	0.12	−0.19 to 0.44	.44
	3 months	4.3	4.0 to 4.7	4.1	3.7 to 4.4	−0.27	−0.60 to 0.052	.1
**Formal**								
	Baseline^a^	3.3	3.0 to 3.7	3.3	3.0 to 3.7	—	—	—
	1 month	2.8	2.4 to 3.1	2.8	2.4 to 3.1	0.0042	−0.25 to 0.26	.97
	3 months	3	2.6 to 3.3	2.8	2.8 to 3.2	−0.20	−0.45 to 0.054	.12
**Web-based/phone**								
	Baseline^a^	3.7	3.4 to 4.0	3.7	3.4 to 4.0	—	—	—
	1 month	3.7	3.3 to 4.1	3.7	3.6 to 4.1	0.031	−0.29 to 0.35	.85
	3 months	3.9	3.5 to 4.3	3.7	3.3 to 4.1	−0.21	−0.54 to 0.13	.23
**None (do not seek help from anyone)**								
	Baseline^a^	3.3	2.9 to 3.8	3.3	2.9 to 3.8	—	—	—
	1 month	2.2	1.7 to 2.7	2.4	1.9 to 2.9	0.17	-0.26 to 0.60	.45
	3 months	2.2	1.8 to 2.7	2.2	1.7 to 2.7	-0.047	-0.45 to 0.36	.82

^a^Estimated mean (95% CI) of baseline outcome for the two study arms are the same because they were constrained to be equal in the mixed-effects model.

^b^Not applicable.

***Link* Use and Satisfaction**

Of the 205 people randomized to the intervention arm, 160 (160/205, 78%) visited the *Link* website and 159 (159/160, 99%) moved beyond the first page. At all 3 follow-up time points, a greater proportion of intervention participants reported the information they found with their respective search strategies helpful and felt surer of themselves compared with the control arm (Table 5). At 1 and 3 months, a greater proportion of participants in the intervention arm reported they had found treatment for their problems compared with the control arm participants. Young people in the intervention arm at the immediate and 3-month time points were more likely to feel that they had been guided to an appropriate service, although this was not evident at 1-month.

**Table 5:** Satisfaction with the search strategies used by study arm for each follow-up time point (count and percentage of participants in the intervention, n=205, and control arm, n=208, who Strongly agreed or agreed to each item).

Benefit of search strategy	Immediate		1 month		3 months	
	*Link*, n (%)	Control, n (%)	*Link*, n (%)	Control, n (%)	*Link*, n (%)	Control, n (%)
Search helped my mental health decisions	90 (43.9)	101 (48.6)	59 (28.8)	54 (26.0)	82 (40.0)	78 (37.5)
I found helpful information	125 (61.0)	105 (50.5)	71 (34.6)	60 (28.8)	91 (44.4)	69 (33.2)
I understood the information	140 (68.3)	130 (62.5)	84 (41.0)	90 (43.3)	97 (47.3)	89 (42.8)
My questions were answered	91 (44.4)	79 (38.0)	40 (19.5)	42 (20.2)	59 (28.8)	58 (27.9)
I found treatment for problems	60 (29.3)	58 (27.9)	36 (17.6)	30 (14.4)	60 (29.3)	49 (23.6)
My symptoms/problems improved	52 (25.4)	66 (31.7)	39 (19.0)	45 (21.6)	70 (34.1)	62 (29.8)
I was guided to appropriate services	98 (47.8)	87 (41.8)	43 (21.0)	48 (23.1)	77 (37.6)	57 (27.4)
I felt surer of myself	81 (39.5)	67 (32.2)	55 (26.8)	46 (22.1)	83 (40.5)	64 (30.8)
My mood was more positive	81 (39.5)	78 (37.5)	52 (25.4)	54 (26.0)	83 (40.5)	77 (37.0)
Searching helped me understand my problems better	88 (42.9)	98 (47.1)	65 (31.7)	63 (30.3)	87 (42.4)	68 (32.7)

### Help-Seeking Strategy After Randomization

Help-seeking results were difficult to interpret because of missing responses to these questions (percentage with missing responses: 15% at immediate time point, 34% at 1-month, and 33% at 3-month follow-up; Multimedia Appendix 5). Of those who responded, the proportion of young people who reported they did not need help across time points was less than 3%. The majority of the participants reported using at least 1 search strategy, with a greater percentage in the intervention arm compared with the control arm at each follow-up time (Multimedia Appendix 5; Table 1). Of those who did seek help, at the immediate time point (up to 2 weeks postrandomization), more young people in the intervention arm used one or more websites or Web-based services to seek help, compared with the control arm (33.5% vs 15.1%), and fewer of the intervention arm used formal (19.8% vs 35.5%) or informal (18.0% vs 27.1%) sources of support (Multimedia Appendix 5; Table 2). Numbers of young people seeking help via phone lines were small in both the arms across all time points. Help-seeking appeared less frequent at both 1- and 3-month follow-up points than immediately after randomization for young people in both the study arms, with young people from the intervention arm more likely to use Web-based sources of help (website/Web-based service and/or other Web-based method) and young people from the control arm more likely to seek help from formal and informal sources of support.

## Discussion

### Principal Findings

This study tested whether *Link*, a website designed to guide young people to appropriate Web-based and computer-based sources of mental health information and care, was effective in increasing psychological well-being and reducing barriers to seeking help for mental health problems. Our results showed that *Link* did not increase PA immediately post intervention compared with usual search strategies. Instead, we found that young people using *Link* and those using their usual search strategies had a similar increase in PA of approximately 30% between baseline and 3 months.

There was, however, a greater reduction in mean NA immediately postintervention and at 1-month for the young people using *Link* compared with the usual search strategies with confidence intervals at 1 month including the hypothesized clinically important value of 2.7, indicating that *Link* did have a short-term benefit in reducing NA. The difference between the arms diminished at 3 months. NA reflects self-reported stress, poor coping, and frequency of negative events; low scores for NA indicate a state of calmness and serenity [27]. The results of this study suggest that NA might be a better measure of immediate benefit and an indicator of any harms of using an intervention to facilitate help-seeking.

There was no difference in general psychological distress between the two arms. Instead, mean K10 scores remained high (ie, >23) for both the arms over time. High levels of distress reported at study entry may indicate that young people with mental health problems are interested in Web-based tools to facilitate help-seeking. Improvement in K10 score might only be expected once the young person was in a therapeutic intervention rather than in the seeking help phase. Higher satisfaction scores among the intervention arm suggests that young people found a youth-focused tool, such as *Link*, to be acceptable.

There was no change in either arm for participants’ perceptions of the barriers or intentions to seek help for mental health problems. However, both arms ranked that they were less likely to not seek help from anybody at 1 and 3 months postintervention than they were at baseline.

Importantly, the economic evaluation of *Link* found that there were quality of life improvements and lower costs in the *Link* arm compared with the control arms [30]. From an economic viewpoint *Link* may be a more efficient use of resources.

### Comparisons With Previous Studies

Recent studies confirm that young people with higher mental health needs are prepared to engage with Web-based strategies to seek help [41,42]. Other digital mediums have been investigated for their potential to address the health care needs of young people. Social media supported interventions have been shown to support weight loss in overweight adolescents [43] and smoking cessation in young adults [44]. A UK study of young people with chronic health conditions found that digital communication was valued by the young people and can assist re-engagement with their clinical specialist teams [45]. Digital storytelling may also have merit as an engagement strategy for health services to use with young people [46]. A recently published Australian study showed that marginalized young people used technology to explore options for their health care and recognized the potential of technology in making the health care system easier for them to navigate and engage with [47]. The value of a tool such as *Link,* specifically designed to promote help-seeking, is not only in facilitating awareness of the types of reputable services available to adolescents when they do need to access them but also in providing information on other issues known to cause access barriers, such as costs, what to expect when visiting the service, and confidentiality, alongside peer stories about the benefits of health care access and tips for well-being.

A study on young people accessing a Web-based mental health support service in Australia (eheadspace) found that the youth had high to very high levels of psychological distress but were at an earlier stage of illness than those presenting to their face-to-face service, which might explain our finding that young people using *Link* were less likely than the control arm to prefer formal sources of mental health care [48]. The potential utility of technology, such as social media, to address health issues affecting young people [43,44] needs to be balanced against other recent studies on potential risks of the internet for adolescents with mental health disorders [49]. The ethical implications of using digital technologies in clinical interactions with young people are only beginning to be explored [50] and include such considerations as how best to promote autonomy in patient’s control over their health care versus dependence on the technology and maintaining confidentiality of interactions. A program such as *Link* is not a clinical tool but a health service navigation tool and the users remain anonymous. However, to increase the chance that young people in need find the tool at a time that would most benefit them, nonclinical Web-based mental health information services might embed a pathway to *Link* from their information pages on mental health issues, so that users can be directed to a range of support options based on their level of distress and support preferences. Furthermore, social media could also be engineered to recognize postings on emotional distress from young people and feed a posting about *Link* to these individuals; this type of intelligence could capture those who would otherwise not proactively seek help. Attitudes to this level of social media artificial intelligence have not yet, to our knowledge, been explored nor has the effectiveness of this approach in promoting mental health help-seeking.

The results of our final outcome trial are timely, as experts urge for consideration of robust policy frameworks to ensure Web-based supports for the mental health of young people are effective, appropriate, and engaging [51]. Our work is timely also because of the pending results of a trial of a similar intervention in Canada, ThoughtSpot, co-designed with young people to enable postsecondary school young people to access mental health support services [52,53]. Given the paucity of evidence for Web-based help-seeking interventions [16], the results of our trial and the ThoughtSpot trial will be important to compare in building our understanding of mental health help-seeking interventions and the degree to which they are effective and efficient.

### Strengths and Limitations

Strengths of the trial include increased precision of estimates as the retention was at least 70% at 3-month follow-up, which was higher than what had been assumed for the sample size estimation. There were also similar withdrawal rates between arms and comparable characteristics between those who withdrew and those who completed the study. Intervention and control arms were well balanced with regard to baseline characteristics and outcome measures were stratified by factors assumed to be associated with the outcome and intervention (gender, recent Web-based mental health searching, and K10 score), demonstrating good internal validity.

A limitation of the study is that the primary outcome, positive PANAS score, was self-reported. To respond accurately, participants must interpret the questions correctly, be aware of their emotional state and feelings, and not be influenced by social desirability bias. Participants were also not blind to whether they received the intervention or not, which might have led to response bias. In addition, as our trial recruited on the Web, the control arm condition of prompting participants to use usual help-seeking strategies might have meant that even control participants used Web-based modalities to seek help, which were encompassing of more conditions than *Link*, a similar issue to what we found in our pilot when we directed control arm participants to Google. This might account for the improvements in PA also seen in the control arm. Although there are more missing values, our data on help-seeking strategies postrandomization (Multimedia Appendix 5) suggest that just under 50% of the control arm seemed to use Web-based sources for help-seeking. Participants in both arms needed to rely on accurate recall when asked about their help-seeking strategies in each follow-up survey; however, recall time periods were short (ie, 2 weeks, 1 month, and 3 months).

The inclusion criteria were broad; however, our findings can only be generalized to young Australians aged 18 to 25 years who use Facebook, Gumtree, and/or Google to search for mental health related services or topics.

### Conclusions

Searching the Web for mental health services and information is common among young people. The process of being prompted to seek mental health information and services appears to improve mood and increase help-seeking intentions among young people, regardless of whether they use a dedicated Web-based youth-focused tool, such as *Link*, or their usual search strategies, which may also include online. However, young people report greater satisfaction using tools designed specifically for them, which may encourage future help-seeking. The ability of Web-based tools to match mental health need with appropriate care should be explored further.

## Appendix

Multimedia Appendix 1 Google and Facebook advertisements.

Multimedia Appendix 2 Screenshots showing Link.

Multimedia Appendix 3 Sensitivity analyses for the missing data assumption.

Multimedia Appendix 4 Baseline characteristics of participants who withdrew by intervention and control arms.

Multimedia Appendix 5 Help-seeking strategies used.

Multimedia Appendix 6 CONSORT-EHEALTH checklist (V 1.6.1).

## Abbreviations

BASH: Barriers to Adolescents Seeking Help
GHSQ: General Help-Seeking Questionnaire
K10: Kessler psychological distress scale
NA: Negative Affect
PA: Positive Affect
PANAS: Positive and Negative Affect Schedule
SMS: Short Message Service
