# Supplementary material for: Effectiveness of a Mental Health Service Navigation Website (Link) for Young Adults: Randomized Controlled Trial
Source: JMIR Ment Health. 2019 Oct 17;6(10):e13189. doi: 10.2196/13189 (PMC6913099; doi:10.2196/13189)
Supplement: Multimedia Appendix 1 [file mental_v6i10e13189_app1.pdf]

Appendix X. Google and Facebook advertisements

Based upon previous research investigating Ads for Facebook, Google and Gumtree, four variations of ads are recommended to recruit participants:

1. One framed in the negative

   Google
   Not coping?
   www.linkurl.edu.au
   We want to know how you find help.
   Join our study today.

   Facebook
   Stressed out?
   www.linkurl.edu.au
   Join our study to help us find out how you seek help with personal or emotional problems.

2. Ones talking about seeking help

   Google
   Are you ok?
   www.linkurl.edu.au
   Dealing with emotional problems?
   Join our study and get support.

   Facebook
   Feeling crap?
   www.linkurl.edu.au
   We are looking for volunteers who need help with personal or emotional problems.

3. Ones talking about the participant (tell us about yourself)

   Google
   Tell us about yourself
   www.linkurl.edu.au
   Get help with personal problems.
   Join our study today.

   Facebook
   Tell us about yourself
   www.linkurl.edu.au
   Join our study about how young adults find help for personal or emotional problems.

4. Ones framed in an altruistic way

   Google

Are you 18-25?
www.linkurl.edu.au
Help us support young Australians.
Join our study today.

Facebook

Support
www.linkurl.edu.au
Join our study and help us understand and support the mental health of young Australians.
