# Supplementary material for: Effectiveness of a Mental Health Service Navigation Website (Link) for Young Adults: Randomized Controlled Trial
Source: JMIR Ment Health. 2019 Oct 17;6(10):e13189. doi: 10.2196/13189 (PMC6913099; doi:10.2196/13189)
Supplement: Multimedia Appendix 2 [file mental_v6i8e13189_app2.pdf]

LOGOUT | EMERGENCY HELP

LINK

LINK HELPS YOU WORK OUT WHAT'S GOING ON, TO CONNECT YOU TO FREE AND/OR PAID SUPPORTSERVICES AND OFFERS PRACTICAL TIPS YOU CAN USE TODAY.

Are you 18-25, going through a tough time and need some help?
Simply tell us what's going on, how it's impacting your life and what kind of support you need.

ABOUT US

WHAT'S GOING ON FOR YOU?

GET STARTED

LOGOUT | EMERGENCY HELP

SELECT ONE

I AM...

01

SELF-HARMING

02

HAVING PROBLEMS WITH PEOPLE CLOSE TO ME

03

OFTEN FEELING STRESSED, WORRIED OR DOWN

04

BEING BULLIED ONLINE, AT SCHOOL OR AT WORK

05

RELYING ON ALCOHOL OR DRUGS TO COPE

07

[illegible]

NEXT

LOGOUT | EMERGENCY HELP

IMPACT ON ME

HOW MUCH IS IT AFFECTING YOU?

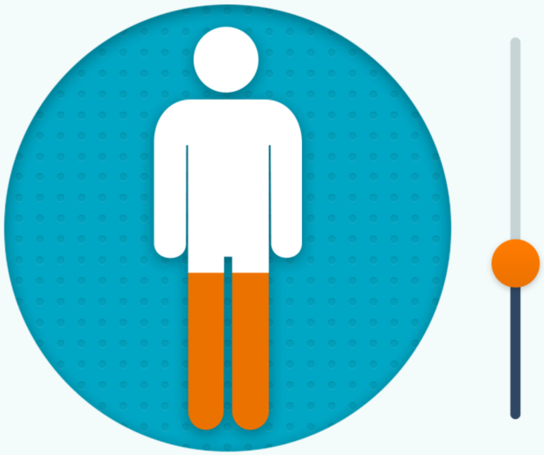

IT'S CRAP BUT I'M OK

NEXT

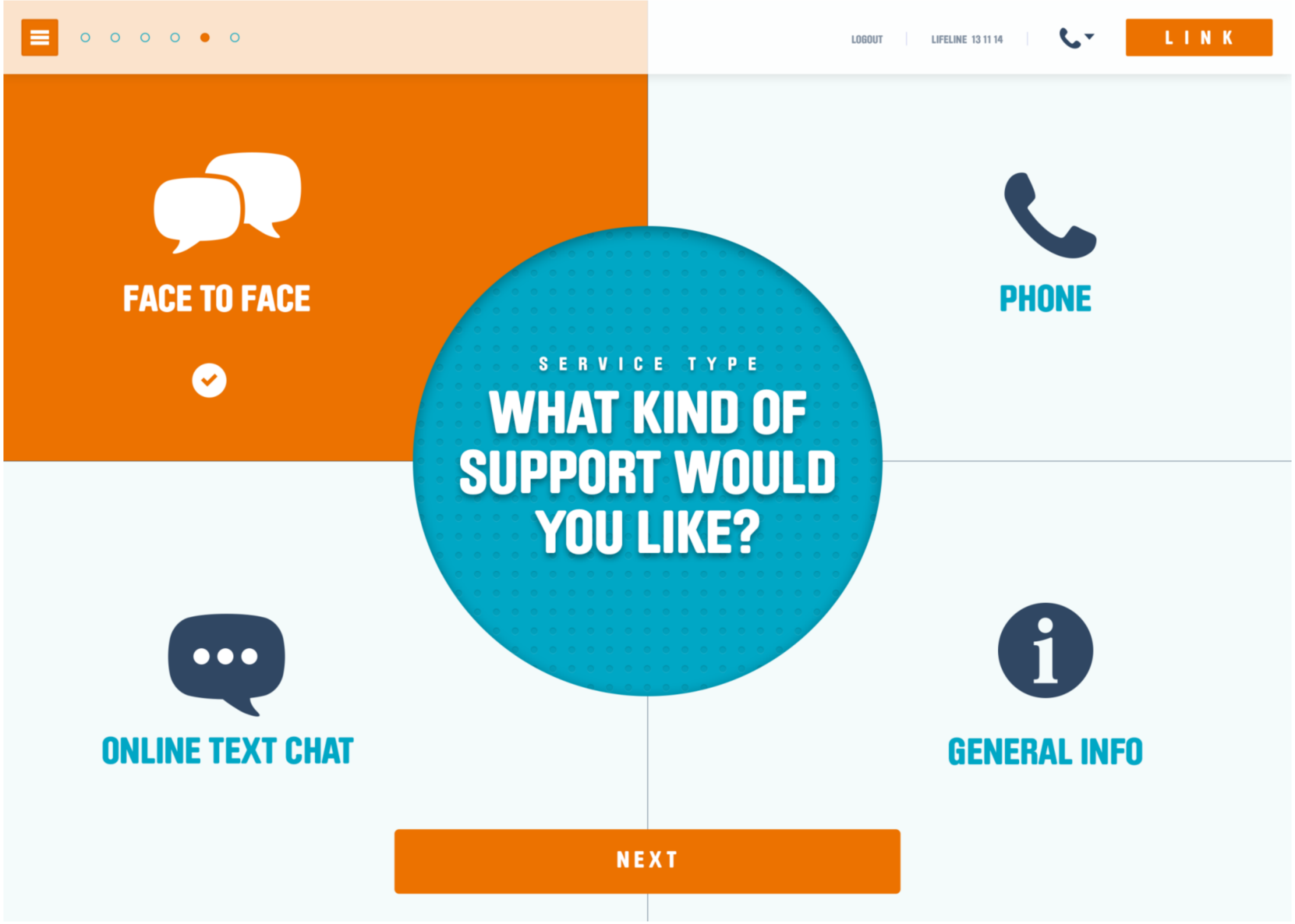
LOGOUT
LIFELINE 13 11 14
LINK
FACE TO FACE
PHONE
SERVICE TYPE
WHAT KIND OF SUPPORT WOULD YOU LIKE?
ONLINE TEXT CHAT
GENERAL INFO
NEXT

RECOMMENDATIONS

I'M OFTEN FEELING STRESSED, WORRIED OR DOWN

Sometimes life gets you down and you just can't find a way to get over it. Everyone feels worry, fear or stress at times but if these feelings start to take over, it's a good idea to talk to somebody.

PRACTICAL TIPS TO TRY

01. EXERCISE

It's probably the last thing on your mind, but getting sweaty can help manage stress, anxiety and depression.

02. BUILD A TOOLKIT

Find some deep breathing and relaxation techniques you can use when things get tough.

03. PRACTICAL STUFF

Deal with issues you might be avoiding, one at a time. Putting them off can feed anxiety.

"It's hard stepping out of your comfort zone. So hard in fact, I couldn't help but wonder why people ever do it. But this year, I think I may have just worked it out..."

GO TO REACHOUT.COM

LINK RECOMMENDS:

What's this?

RECOMMENDED SERVICES

Find out why

General Practitioner (GP)

A local doctor who treats a range of physical and mental health issues.

Cost: Medicare

Find nearest

Psychologist

A trained doctor who deals with mental health and helps you cope in tough times.

Cost: Medicare

Find nearest

headspace

National Youth Mental Health Foundation

A community centre offering face-to-face support with counsellors and other medical professionals.

Cost: Free

View website

General Practitioner (GP)

AVAILABILITY:

Hours and wait times can vary—call or visit their website to check.

COST:

Some charge privately, but most bulk bill through Medicare. Find out more

CONFIDENTIAL

Find out more about your rights and confidentiality

HOW IT WORKS

GPs are experts in dealing with all kinds of physical and mental health issues, including:

- sleeping problems
- feeling down or lacking energy
- issues with weight, exercise or food
- relationship problems
- drugs and alcohol.

GPs can also refer you to a specialist, like a psychologist or counsellor, if needed.

WHAT TO EXPECT

- GPs have different hours and waiting times. If you're not sure, call and ask or visit their website if they have one.
- You will probably need to make an appointment if it's at a smaller practice, however most medical centres normally let you walk in without an appointment—ask about expected wait time.
- You don't have to tell the receptionist what the problem is, just say you want to see a doctor and specify male or female if you have a preference.
- Depending on your situation, you can expect the GP to ask about your health and what's bothering you.
- They may do a physical check-up, either generally or in the area that's worrying you, but they can't do this without your permission.
- Try writing down your situation or any questions you have before you go, so you don't forget.

CALL NOW

FIND A GP NEAR YOU

LOOK AT ANOTHER ISSUE

LOGOUT | EMERGENCY HELP

SELECT ONE

EMERGENCY CONTACTS

LIFELINE

13 11 14

For support and advice in a personal crisis.

CONTACT

Phone counselling 24/7

Online web chat, check site for more details **www.lifeline.org.au**

SUICIDE CALL BACK SERVICE

1300 65 94 67

Support if you, or someone you know, is feeling suicidal.

CONTACT

Phone counselling 24/7

Online web chat, check site for more details **www.suicidecallbackservice.org.au**

ARE YOU IN DANGER?

000

If you, or someone you are with, is in immediate danger

OR

go to your nearest hospital emergency department.

NEXT

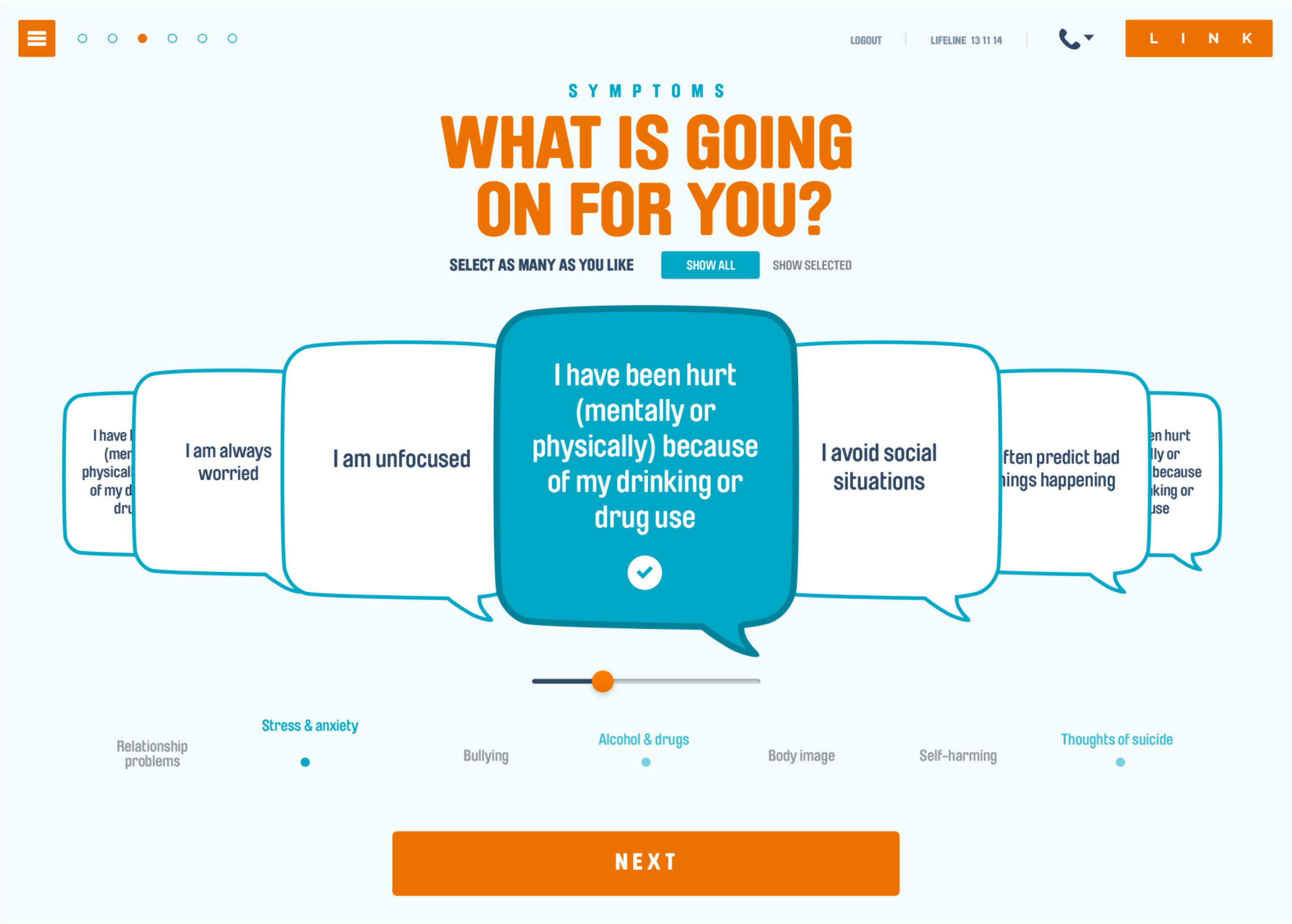
LOGOUT
LIFELINE 13 11 14
L I N K
S Y M P T O M S
WHAT IS GOING ON FOR YOU?
SELECT AS MANY AS YOU LIKE
SHOW ALL
SHOW SELECTED
I am always worried
I am unfocused
I have been hurt (mentally or physically) because of my drinking or drug use
I avoid social situations
Relationship problems
Stress & anxiety
Bullying
Alcohol & drugs
Body image
Self-harming
Thoughts of suicide
NEXT

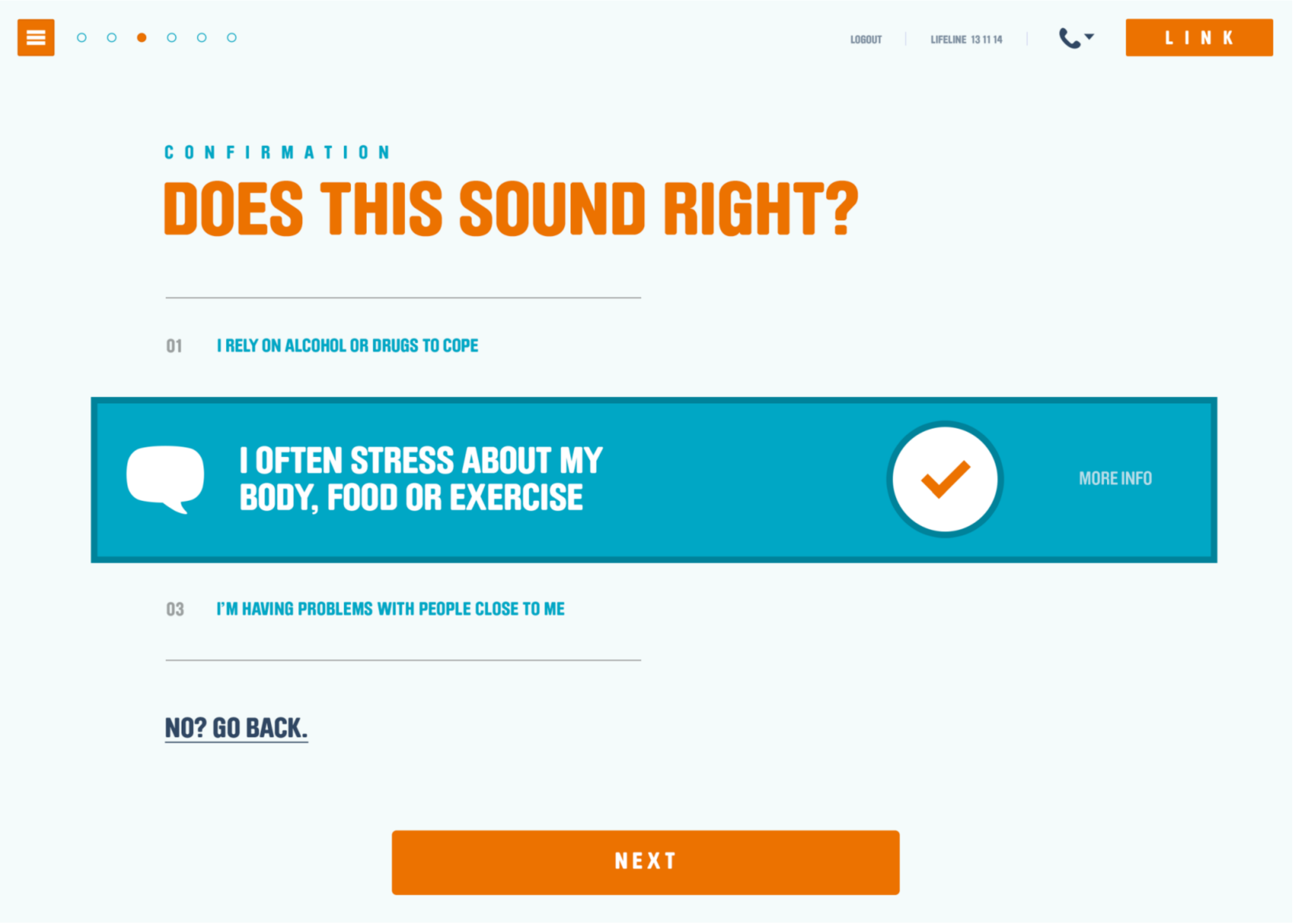
LOGOUT
LIFELINE 13 11 14
LINK
CONFIRMATION
DOES THIS SOUND RIGHT?
01 I RELY ON ALCOHOL OR DRUGS TO COPE
I OFTEN STRESS ABOUT MY BODY, FOOD OR EXERCISE
MORE INFO
03 I'M HAVING PROBLEMS WITH PEOPLE CLOSE TO ME
NO? GO BACK.
NEXT

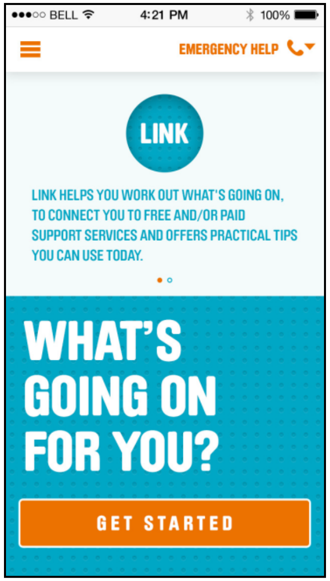
●●●○○ BELL
4:21 PM
100%
EMERGENCY HELP
LINK
LINK HELPS YOU WORK OUT WHAT'S GOING ON, TO CONNECT YOU TO FREE AND/OR PAID SUPPORT SERVICES AND OFFERS PRACTICAL TIPS YOU CAN USE TODAY.
WHAT'S GOING ON FOR YOU?
GET STARTED

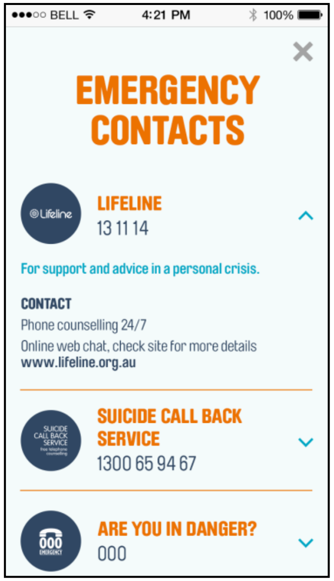
●●●○○ BELL
4:21 PM
100%
EMERGENCY CONTACTS
LIFELINE
13 11 14
For support and advice in a personal crisis.
CONTACT
Phone counselling 24/7
Online web chat, check site for more details
www.lifeline.org.au
SUICIDE CALL BACK SERVICE
1300 65 94 67
ARE YOU IN DANGER?
000

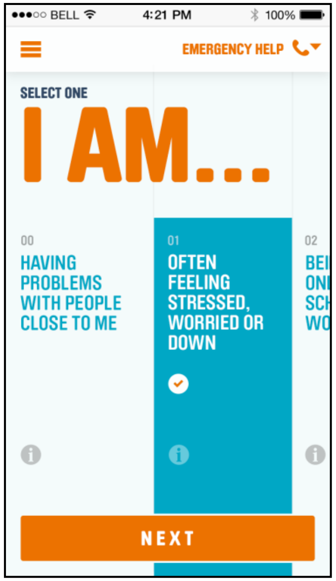
●●●○○ BELL
4:21 PM
100%
EMERGENCY HELP
SELECT ONE
I AM...
00
HAVING PROBLEMS WITH PEOPLE CLOSE TO ME
01
OFTEN FEELING STRESSED, WORRIED OR DOWN
02
NEXT

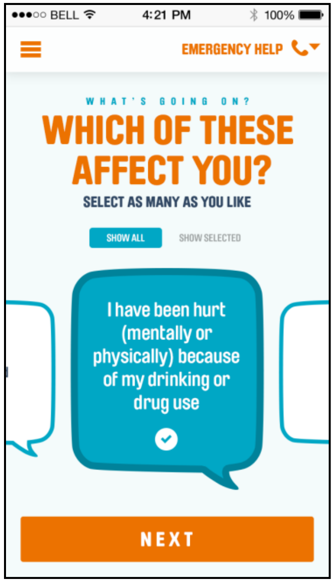
●●●○○ BELL
4:21 PM
100%
EMERGENCY HELP
WHAT'S GOING ON?
WHICH OF THESE AFFECT YOU?
SELECT AS MANY AS YOU LIKE
SHOW ALL
SHOW SELECTED
I have been hurt (mentally or physically) because of my drinking or drug use
NEXT

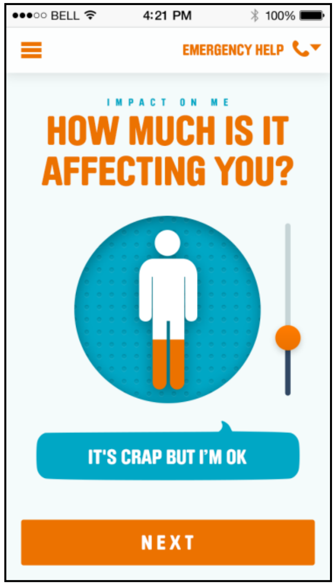
●●●○○ BELL
4:21 PM
100%
EMERGENCY HELP
IMPACT ON ME
HOW MUCH IS IT AFFECTING YOU?
IT'S CRAP BUT I'M OK
NEXT

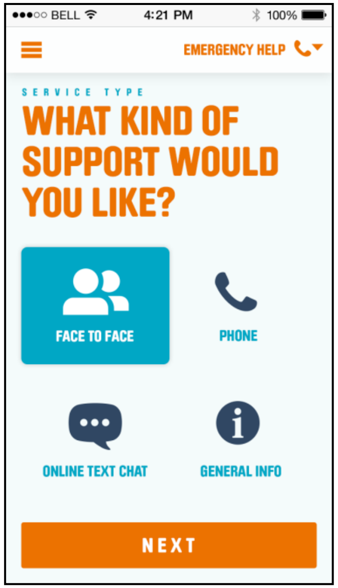
●●●○○ BELL
4:21 PM
100%
EMERGENCY HELP
SERVICE TYPE
WHAT KIND OF SUPPORT WOULD YOU LIKE?
FACE TO FACE
PHONE
ONLINE TEXT CHAT
GENERAL INFO
NEXT

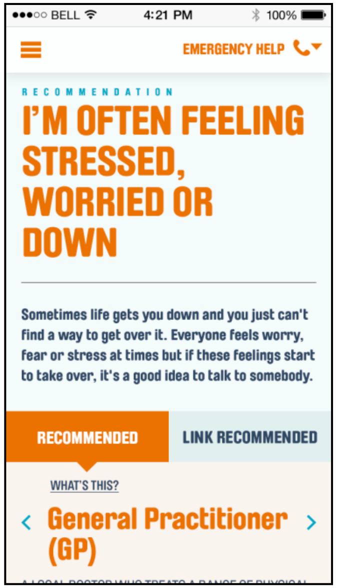
●●●○○ BELL
4:21 PM
100%
EMERGENCY HELP
RECOMMENDATION
I'M OFTEN FEELING STRESSED, WORRIED OR DOWN
Sometimes life gets you down and you just can't find a way to get over it. Everyone feels worry, fear or stress at times but if these feelings start to take over, it's a good idea to talk to somebody.
RECOMMENDED
LINK RECOMMENDED
WHAT'S THIS?
General Practitioner (GP)
