# Supplementary material for: Effectiveness of a Mental Health Service Navigation Website (Link) for Young Adults: Randomized Controlled Trial
Source: JMIR Ment Health. 2019 Oct 17;6(10):e13189. doi: 10.2196/13189 (PMC6913099; doi:10.2196/13189)
Supplement: Multimedia Appendix 3 [file mental_v6i8e13189_app3.pdf]

Multimedia Appendix 3: Sensitivity analyses for the missing data assumption

The results from the linear mixed effects regression model used in the main analysis to estimate the effect of the *Link* program on positive affect score assumes that data are missing at random (MAR). Under this assumption, the difference between the mean positive affect score of the missing data and the mean of the positive affect scores of the observed data δ, is zero. To test the robustness of the missing data assumption for the regression analysis, a sensitivity analysis was performed using a pattern mixture model, whereby plausible values for δ ranging between -10 and 10 in increments of 1 were considered. Positive values of δ indicate that on average, participants with missing data have higher (better) positive affect scores than observed participants and negative values of δ assume participants with missing data have lower (worse) mean positive affect scores than observed participants.

Appendix 3 Figure 1 plots the estimated intervention effect with respective 95% confidence intervals for the two study arms for selected parameter values δ on the x-axis at two weeks (immediately). A horizontal reference line is plotted at zero on the y-axis, where positive values of the estimated intervention effect indicate that the mean positive affect score is the intervention arm is higher (better) than the control arm and negative values indicate that the control arm have higher (better) mean positive affect scores than the control arm. We repeated the sensitivity analysis for the missing data assumption for positive affect scores at one and three months follow-up and negative affect scores at immediate, one and three months (results shown in Appendix Figures 1-6).

Interpretation of the results

The estimated intervention effect for positive affect score immediately (Appendix 3 Figure 1), one- and three-months post-intervention remained relatively constant across the range of values of δ for data departing from missing at random in the same way in both arms. Since the

proportion of missing data was similar in the two study arms, the effects of departures from the missing at random assumption were similar in the intervention and control arms and any variation in effect was mainly due to differences in δ between study arms. Therefore, for the study conclusions to change, departures from the missing at random assumption would need to differ in the two study arms or be present in the intervention arm alone or the control arm alone. At three months follow-up for example, the mean PA score of the missing data would need to be at least three points higher than the mean of the observed data in the intervention arm alone for the conclusions to change or at least three points lower in the control arm for the conclusions to change. Whilst this may seem plausible given the standard deviation for the PA score at three months is nine, the characteristics and baseline PA scores of those with missing data are similar between the study arms. It therefore seems unlikely that departures from the missing at random assumption would occur differently in the two study arms.

For the negative affect score, the sensitivity analysis showed that the mean of the missing data needed to be seven points lower than the mean of the observed data in both arms or different by two points in the intervention or control arms alone for the study conclusions to change. Again, it seems implausible that data would depart from missing at random in one arm only as characteristics of those with missing negative affect data are similar between the two study arms.
