# Supplementary material for: Effectiveness of a Mental Health Service Navigation Website (Link) for Young Adults: Randomized Controlled Trial
Source: JMIR Ment Health. 2019 Oct 17;6(10):e13189. doi: 10.2196/13189 (PMC6913099; doi:10.2196/13189)
Supplement: Multimedia Appendix 4 [file mental_v6i8e13189_app4.pdf]

Multimedia Appendix 4: Baseline characteristics of participants that withdrew by intervention and control arms[a,b]

	Link **n = 59**			**Control n = 64**		
	**n**	**(%)**	**n (%) of missing responses** [c]	**n**	**(%)**	**n (%) of missing responses** [c]
Female	49	(83.1)	0 (0)	54	(84.4)	0 (0)
Neither working nor studying	4	(8.2)	10 (17.0)	6	(10.7)	8 (12.5)
Socio-economic advantage[d]	29	(61.7)	12 (20.3)	37	(77.1)	16 (25.0)
Rural[d]	18	(38.3)	12 (20.3)	12	(25.0)	16 (25.0)
English not spoken at home	9	(15.3)	0 (0)	9	(14.1)	0 (0)
Aboriginal or Torres Strait Islander	1	(1.8)	3 (5.1)	4	(6.5)	3 (4.7)
**Highest Education level**			0 (0)			0 (0)
Did not complete secondary school	3	(5.1)		7	(10.9)	
Completed/partially completed years 11/12	34	(57.6)		33	(51.6)	
Certificate or diploma	10	(17.0)		13	(20.3)	
Undergraduate degree	10	(17.0)		11	(17.2)	
Post graduate degree, masters or PhD	2	(3.4)		0	(0)	
**Mental health rating (K10)**			0 (0)			0 (0)
No illness/problems	4	(6.8)		7	(10.9)	
Some symptoms but no disease	15	(25.4)		19	(29.7)	
Minor illness	16	(27.1)		14	(21.9)	
Moderate illness	18	(30.5)		20	(31.1)	
Severe illness	6	(10.1)		4	(6.3)	
**Self-reported issues**[d]			0 (0)			0 (0)
**Number of issues reported**						
None	7	(11.9)		6	(9.4)	
One	15	(25.4)		16	(25.0)	
Two or more	37	(62.7)		42	(65.6)	
**Issue reported by participants** [e]						
Often stressed, worried or down	45	(76.3)		49	(76.6)	
Often stressing about body, food or exercise	28	(47.5)		38	(59.4)	
Worried about my drug or alcohol use	4	(6.8)		5	(7.8)	
Harming myself	4	(6.8)		5	(7.8)	

Thinking about ending my life	9	(15.3)		10	(15.6)	
Being bullied online, school or work	2	(3.4)		1	(1.6)	
Having problems with people close to me	12	(20.3)		18	(28.1)	
**Primary and Secondary Outcomes**			0 (0)			0 (0)
Positive Affect– Mean (SD)	23.9	(8.4)		24.4	(7.9)	
Negative affect– Mean (SD)	20.4	(7.8)		22.5	(10.0)	
Psychological distress (K10)- Mean (SD)	28.3	(9.5)		27.9	(10.2)	
Barriers to Seeking Help (BASH)- Mean (SD)	37.9	(9.7)		37.9	(9.2)	

SD = Standard deviation

[a]Percentages may not sum to 100% due to rounding and totals may vary due to missing responses. [b]Counts and percentages presented unless otherwise stated. [c]Number of missing responses presented as count and % of total allocated to intervention arm (n=205) and control arm (n=208), respectively. [d]Index of Relative Socio-Economic Advantage and Disadvantage (IRSAD) Australian Bureau Statistics. [e]Sub-categories are not mutually exclusive.
