# Supplementary material for: Effectiveness of a Mental Health Service Navigation Website (Link) for Young Adults: Randomized Controlled Trial
Source: JMIR Ment Health. 2019 Oct 17;6(10):e13189. doi: 10.2196/13189 (PMC6913099; doi:10.2196/13189)
Supplement: Multimedia Appendix 5 [file mental_v6i8e13189_app5.pdf]

Multimedia Appendix 5: Help-seeking strategies used

Table 1 below summarises the responses to the ‘Strategy used’ question for help-seeking by arm and follow up time point. Participants responses to the question were categorised as "No strategy used", "Did not need help" or "Help-seekers" if they used at least one or more of the following help-seeking search strategies: "General search" (e.g. google, Link), "Website/ online service"(e.g. beyondblue, Reachout, Headspace/eHeadspace), "Other online strategy" (e.g. online [not specified], online forums or chatrooms), "Formal sources of support" (e.g. GP, psychologist, counsellor, school/uni support), "Informal sources of support" (e.g. talk to family/friends/partner/colleague, religion support), "Phone line" (e.g. lifeline, kids helpline), or "Other sources of support" (e.g. meditation/relaxation/mindfulness, books, information [not specified]). Table 2 shows the count and percentage of participants classified as help-seekers in Table 1 for each of the categories listed above. Results are presented as ’None’ or ‘1 or more’ results.

Appendix 5 Table 1. Participants not needing or not seeking help by intervention and control arm for each follow-up time period[a]

	**Immediate time point**		**1-month follow-up**		**3-month follow-up**	
	Link	**Control**	***Link***	**Control**	***Link***	**Control**
**Number who responded to question**	**(n=173*)***	**(n=177)**	**(n=135)**	**(n=136)**	**(n=143)**	**(n=132)**
**Help-seeking**	**n (%)**	**n (%)**	**n (%)**	**n (%)**	**n (%)**	**n (%)**
**No strategy used**	5 (2.9)	11 (6.2)	11 (8.1)	22 (16.2)	8 (5.6)	15 (11.4)
**Did not need help**	1 (0.5)	0 (0)	1 (0.7)	1 (0.7)	3 (2.1)	3 (2.3)
**Help-seekers[b]**	167 (96.5)	166 (93.8)	123 (91.1)	113 (83.7)	132 (92.3)	114 (86.4)

[a] Number allocated to intervention arm (n=205) and control arm (n=208)

[b]Total number of respondents who reported using one or more help-seeking strategies

Appendix 5 Table 2. Strategies for Help-seeking used post-randomisation by arm[a,b]

	Immediate time point				1-month follow-up				3-month follow-up			
	Intervention		Control		Intervention		Control		Intervention		Control	
	**n**	**%**	**n**	**%**	**n**	**%**	**n**	**%**	**n**	**%**	**n**	**%**
**General search**												
None	129	77.2	124	74.7	116	94.3	106	93.8	123	93.2	109	95.6
1 or more	38	22.8	42	25.3	7	5.7	7	6.2	9	6.8	5	4.4
**Website/online service**												
None	111	66.5	141	84.9	101	82.1	101	89.4	119	90.2	102	89.5
1 or more	56	33.5	25	15.1	22	17.9	12	10.6	13	9.8	12	10.5
**Other online strategy**												
None	116	69.5	110	66.3	81	65.9	87	77.0	98	74.2	86	75.4
1 or more	51	30.5	56	33.7	42	34.1	26	23.0	34	25.8	28	24.6
**Formal sources of support**												
None	134	80.2	107	64.5	89	72.4	74	65.5	88	66.7	67	58.8
1 or more	33	19.8	59	35.5	34	27.6	39	34.5	44	33.3	47	41.2
**Informal sources of support**												
None	137	82.0	121	72.9	87	70.7	67	59.3	87	65.9	69	60.5
1 or more	30	18.0	45	27.1	36	29.3	46	40.7	45	34.1	45	39.5
**Phone line**												
None	166	99.4	160	96.4	120	97.6	110	97.3	129	97.7	113	99.1
1 or more	1	0.6	6	3.6	3	2.4	3	2.7	3	2.3	1	0.9
**Other sources of support**												
None	147	88.0	147	88.6	99	80.5	92	81.4	118	89.4	98	86.0
1 or more	20	12.0	19	11.4	24	19.5	21	18.6	14	10.6	16	14.0

[a]Results are presented as a percentage of respondents who reported using a help-seeking strategy per arm at each time point. [b]Strategies used for help-seeking were not mutually exclusive.
