# Supplementary material for: Effectiveness of a Mental Health Service Navigation Website (Link) for Young Adults: Randomized Controlled Trial
Source: JMIR Ment Health. 2019 Oct 17;6(10):e13189. doi: 10.2196/13189 (PMC6913099; doi:10.2196/13189)
Supplement: Multimedia Appendix 6 [file mental_v6i10e13189_app6.pdf]

CONSORT-EHEALTH Checklist V1.6.2 Report

(based on CONSORT-EHEALTH V1.6), available at [http://tinyurl.com/consort-ehealth-v1-6].

Date completed

12/18/2018 8:21:49

by

Lena Sanci

Positive affect and help-seeking effects of a mental health service navigation website for young adults called Link: A randomised controlled trial

TITLE

1a-i) Identify the mode of delivery in the title

"Positive affect and help-seeking effects of a mental health service navigation website for young adults called Link: A randomised controlled trial"

1a-ii) Non-web-based components or important co-interventions in title

There are no non-web-based components to the intervention

1a-iii) Primary condition or target group in the title

"Positive affect and help-seeking effects of a mental health service navigation website for young adults called Link: A randomised controlled trial"

ABSTRACT

1b-i) Key features/functionalities/components of the intervention and comparator in the METHODS section of the ABSTRACT

"To support young people in their help-seeking, we developed an online mental health service navigation website called Link. Link is based on the Theory of Planned Behaviour and connects young people with treatment based on the type and severity of mental health symptoms they report."

1b-ii) Level of human involvement in the METHODS section of the ABSTRACT

It is difficult to include this level of detail within the word limit of an abstract

1b-iii) Open vs. closed, web-based (self-assessment) vs. face-to-face assessments in the METHODS section of the ABSTRACT

Participant recruitment is explained: "Young people, aged between 18 and 25 years, were recruited online from an open access website to participate in a randomised controlled trial." The mechanism of the trial and the nature of the assessment measures are also reported: "Baseline, immediate post-intervention, one-month and three-month surveys were all self-report and administered online."

1b-iv) RESULTS section in abstract must contain use data

Usage data and the uptake of the intervention is reported: "431 young people were recruited into the trial (intervention n = 205; control n = 208) and 78% of those randomised to the intervention arm visited the Link website."

1b-v) CONCLUSIONS/DISCUSSION in abstract for negative trials

"The process of prompting young people to seek mental health information and services appears to improve affective state and increase help-seeking intentions among young people, regardless of whether they use an online dedicated youth-focused tool such as Link or their usual search strategies."

INTRODUCTION

2a-i) Problem and the type of system/solution

"Not knowing where or how to access services, perceived costs and inconvenience in accessing care, and fears of being judged or of breaches in confidentiality, are other important barriers perceived by young people" explains the problem and "Interventions that reduce these barriers and provide a positive experience of help-seeking are needed, particularly interventions that facilitate access to treatments" explains the rationale for the intervention.

2a-ii) Scientific background, rationale: What is known about the (type of) system

"Mental ill-health is a leading health burden affecting one in four young people worldwide " and "Most young people have lived their entire lives in a digital media-saturated world and are highly likely to use the internet to search for health information "

Does your paper address CONSORT subitem 2b?

"The primary objective was to assess the impact of Link on young people's positive affect compared to usual help-seeking strategies immediately post intervention." Secondary objectives (outcomes) are also described in the introduction.

METHODS

3a) CONSORT: Description of trial design (such as parallel, factorial) including allocation ratio

"This was an Australian-based individually randomised controlled trial conducted between 27 November 2014 (first participant recruited) and 4th July 2015 (last follow-up survey completed). All study procedures were conducted online."

3b) CONSORT: Important changes to methods after trial commencement (such as eligibility criteria), with reasons

No changes to the methods occurred after trial commencement. Conducting a pilot feasibility study prior to the main RCT may have assisted in this respect.

3b-i) Bug fixes, Downtimes, Content Changes

This was not applicable in our study

4a) CONSORT: Eligibility criteria for participants

"Participants were eligible if they were aged between 18 and 25 years, living in Australia, and had sufficient English and computer literacy to complete the survey measures and navigate the Link website."

4a-i) Computer / Internet literacy

"Participants were eligible if they were aged between 18 and 25 years, living in Australia, and had sufficient English and computer literacy to complete the survey measures and navigate the Link website."

4a-ii) Open vs. closed, web-based vs. face-to-face assessments:

"A digital marketing company (Profero) were responsible for participant recruitment. The marketing strategy was in English and comprised electronic direct mail, social media, online advertising (Facebook, Gumtree, Google), and snowballing."

4a-iii) Information giving during recruitment

"Young people who met the eligibility criteria and provided informed consent registered for the trial using their email address and a self-generated password." Participants had to be willing to take the additional step of registering for the trial.

4b) CONSORT: Settings and locations where the data were collected

"Participants were eligible if they were aged between 18 and 25 years, living in Australia, and had sufficient English and computer literacy to complete the survey measures and navigate the Link website."

4b-i) Report if outcomes were (self-)assessed through online questionnaires

"All study procedures were conducted online."

4b-ii) Report how institutional affiliations are displayed

Our affiliation is listed as part of the consenting procedures, as is required for ethics approval. It was not used as a marketing strategy.

5) CONSORT: Describe the interventions for each group with sufficient details to allow replication, including how and when they were actually administered

5-i) Mention names, credential, affiliations of the developers, sponsors, and owners

"It (Link) was designed by the research team in conjunction with young people and developed by the software company TigerSpike (https://tigerspike.com/)."

5-ii) Describe the history/development process

"It was designed by the research team in conjunction with young people and developed by the software company TigerSpike (https://tigerspike.com/)."

5-iii) Revisions and updating

No revisions to the online intervention occurred after trial commencement. Conducting a pilot feasibility study prior to the main RCT may have assisted in this respect.

5-iv) Quality assurance methods

This was addressed by conducting a feasibility study prior to the main RCT.

5-v) Ensure replicability by publishing the source code, and/or providing screenshots/screen-capture video, and/or providing flowcharts of the algorithms used

Provided in Figure 1 in the manuscript

5-vi) Digital preservation

Our intervention is no longer accessible.

5-vii) Access

"Email addresses and passwords allowed all participants to login and complete surveys at each wave and use the Link program (intervention arm only)."

5-viii) Mode of delivery, features/functionalities/components of the intervention and comparator, and the theoretical framework

"Link is a self-directed mental health help-seeking service navigation website designed to guide young people to appropriate online and offline sources of mental health information and care." and "It was designed by the research team in conjunction with young people and developed by the software company TigerSpike (https://tigerspike.com/). The theoretical basis and rationale for each feature of Link has been published previously." A lot more detail about the intervention is provided in the manuscript.

5-ix) Describe use parameters

"Users could access Link using either a computer, tablet or smartphone. Intervention participants could use Link as often as they wished throughout the study."

5-x) Clarify the level of human involvement

"Firstly, the user is asked to select from a list of symptoms, expressed in language co-designed by young people, the one that best reflects how they are feeling. The symptoms map to eight domains: anxiety and depression; bullying; alcohol and drug problems; issues with eating, weight and body image; relationship difficulties; suicidal intent; and, self-harm. Secondly, users are asked to rate the degree to which the symptoms are affecting them using an interactive pictorial five-point sliding scale ranging from 1 = 'I'm OK' to 5 = 'It's a huge deal'. Thirdly, they select their service preference, i.e. face-to-face; phone helpline, online chat or email therapy, or online information and self-help. Based on information provided in steps one to three, Link presents three service recommendations from a directory of 31 youth-friendly services."

5-xi) Report any prompts/reminders used

"Individuals who partially completed the baseline survey or did not complete the randomisation process were sent email and SMS reminders four, seven, and 14 days after beginning the enrolment process. Individuals who completed the enrolment process in less than 28 days were considered enrolled in the study." Reminder procedures for the immediate, one- and three-month surveys are also described in the manuscript.

5-xii) Describe any co-interventions (incl. training/support)

"The program also recommends a suitable service modality based upon the severity of the issue. For example, if the user selected 'online information' as a service preference for severe thoughts of self-harm, Link would also suggest a 24-hour telephone helpline."

6a) CONSORT: Completely defined pre-specified primary and secondary outcome measures, including how and when they were assessed

"The primary outcome was mean change in positive affect from baseline to immediate post-intervention in the intervention relative to the control arm." and "Secondary outcomes included PA at all other follow-up points, and the measures described below (Table 1)."

6a-i) Online questionnaires: describe if they were validated for online use and apply CHERRIES items to describe how the questionnaires were designed/deployed

The primary and secondary outcome measures are described in the manuscript. The survey administration is described: " Email addresses and passwords allowed all participants to login and complete surveys at each wave and use the Link program (intervention arm only)." Participants did not have to complete the survey, but the incentives were only available to those who did: "Participants were reimbursed with a AU$15 gift card for completing each of the first two surveys and AU$20 for completing the final survey." The study time frame is described: "This was an Australian-based individually randomised controlled trial conducted between 27 November 2014 (first participant recruited) and 4th July 2015 (last follow-up survey completed).", as is the timing of survey administration: "All outcome measures were collected at one month and three months post-intervention. Positive and negative affect and satisfaction were also measured immediately after the intervention (Table 1)." As previously validated survey items were primarily used to measure the outcomes the survey questions were not randomised or adapted, and the number of questions was not reduced. Participants were able to elect to leave most items blank, although "Checks for valid input data were programmed into QuON, so that only valid survey responses could be entered. " Participants could additionally save the survey and return at a later time to complete it.

6a-ii) Describe whether and how "use" (including intensity of use/dosage) was defined/measured/monitored

"All activity in Link was tracked, recorded and linked to the intervention participants' unique identification number."

6a-iii) Describe whether, how, and when qualitative feedback from participants was obtained

"Both arms were asked about the method they used to seek help in the first two weeks (immediate), one month and three months after randomisation mainly to gain an understanding of methods used by the control arm." This data was recorded as free text responses.

6b) CONSORT: Any changes to trial outcomes after the trial commenced, with reasons

"Participants were eligible if they were aged between 18 and 25 years, living in Australia, and had sufficient English and computer literacy to complete the survey measures and navigate the Link website."

7a) CONSORT: How sample size was determined

7a-i) Describe whether and how expected attrition was taken into account when calculating the sample size

"A sample size estimate of 214 participants (107 per arm) was based on the positive affect scale of the Positive Affect and Negative Affect Scale (PANAS) with a minimal clinically significant difference in mean scores between the two arms of 2.7, assuming a standard deviation of 7.9, 80% power and 5% significance level. To test our primary hypothesis that participants in the intervention arm would on average report an increase in positive affect immediately after using Link compared to participants in the control arm, we based our sample size calculations on a one-tailed independent t-test. Due to the high attrition rates commonly observed in online recruitment, the sample size was inflated by two thirds to 336 young adults (168 participants in each arm). "

7b) CONSORT: When applicable, explanation of any interim analyses and stopping guidelines

"The primary outcome was mean change in positive affect from baseline to immediate post-intervention in the intervention relative to the control arm." and "Secondary outcomes included PA at all other follow-up points, and the measures described below (Table 1)."

8a) CONSORT: Method used to generate the random allocation sequence

"A 32-character unique identification code comprising letters and numbers was assigned to each participant. Participants were randomly allocated to either the intervention or control arm, after completing the baseline measures using a random allocation sequence generated internally by the QuON computer software (The University of Newcastle Australia and Australian National Data Service, 2013).

8b) CONSORT: Type of randomisation; details of any restriction (such as blocking and block size)

"Randomisation was stratified by gender (male, female) and psychological distress (K10 score<20 and K10 score>=20) using random sequences of block sizes of four, six or eight within each stratum and an allocation ratio of 1:1.""

9) CONSORT: Mechanism used to implement the random allocation sequence (such as sequentially numbered containers), describing any steps taken to conceal the sequence until interventions were assigned

"A statistician not involved with the research oversaw this procedure to ensure accuracy and blinding of the research team."

10) CONSORT: Who generated the random allocation sequence, who enrolled participants, and who assigned participants to interventions

"Participants were randomly allocated to either the intervention or control arm, after completing the baseline measures using a random allocation sequence generated internally by the QuON computer software."

11a) CONSORT: Blinding - If done, who was blinded after assignment to interventions (for example, participants, care providers, those assessing outcomes) and how

11a-i) Specify who was blinded, and who wasn't

"Researchers and statisticians involved in the data analysis were blind to the allocation of participants until after data analysis was completed."

11a-ii) Discuss e.g., whether participants knew which intervention was the "intervention of interest" and which one was the "comparator"

"It was not possible to blind participants to the study arm to which they were assigned as the study information stated that they would be asked to look for services either through usual methods or an online program."

11b) CONSORT: If relevant, description of the similarity of interventions

Not applicable

12a) CONSORT: Statistical methods used to compare groups for primary and secondary outcomes

"Linear mixed-effects models with random intercepts were used to estimate differences in mean outcome between the study arms using restricted maximum likelihood estimation at each time-point. Individual participant data were treated as random effects and an unstructured correlation structure was used to account for the repeated measures." The manuscript provides a lot more detail about the statistical analyses.

12a-i) Imputation techniques to deal with attrition / missing values

How missing values were treated is described in the manuscript and the justification for this is included in an Appendix: "Under the fitted linear mixed-effects model, missing data were assumed to be missing at random. A sensitivity analysis was performed using a pattern–mixture model to assess the robustness of this assumption for the PANAS (details provided in Appendix 1)."

12b) CONSORT: Methods for additional analyses, such as subgroup analyses and adjusted analyses

"In a secondary analysis, estimates for all outcomes were also adjusted by whether participants had searched online for mental health services in the two weeks (yes/no) prior to commencement of the study. Residual plots were examined to assess the goodness of fit of the models (results not shown)."

RESULTS

13a) CONSORT: For each group, the numbers of participants who were randomly assigned, received intended treatment, and were analysed for the primary outcome

"Participant flow through the study is shown in Figure 2."

13b) CONSORT: For each group, losses and exclusions after randomisation, together with reasons

"Participant flow through the study is shown in Figure 2."

13b-i) Attrition diagram

"Participant flow through the study is shown in Figure 2."

14a) CONSORT: Dates defining the periods of recruitment and follow-up

These are described in the methods section

14a-i) Indicate if critical "secular events" fell into the study period

This did not apply to our study.

14b) CONSORT: Why the trial ended or was stopped (early)

This did not apply to our study.

15) CONSORT: A table showing baseline demographic and clinical characteristics for each group

"Baseline characteristics of participants are summarised in Table 2."

15-i) Report demographics associated with digital divide issues

"Baseline characteristics of participants are summarised in Table 2."

16a) CONSORT: For each group, number of participants (denominator) included in each analysis and whether the analysis was by original assigned groups

16-i) Report multiple "denominators" and provide definitions

Denominators are reported with results in Tables 3, 4, 5 and 6.

16-ii) Primary analysis should be intent-to-treat

The intention to treat approach was used for the primary analysis and a number of the secondary analyses.

17a) CONSORT: For each primary and secondary outcome, results for each group, and the estimated effect size and its precision (such as 95% confidence interval)

See Tables 4 and 5 for the primary and secondary outcomes.

17a-i) Presentation of process outcomes such as metrics of use and intensity of use

This data is presented in Appendix 2.

17b) CONSORT: For binary outcomes, presentation of both absolute and relative effect sizes is recommended

Not applicable.

18) CONSORT: Results of any other analyses performed, including subgroup analyses and adjusted analyses, distinguishing pre-specified from exploratory

This data is presented in Appendix 1.

18-i) Subgroup analysis of comparing only users

Subgroup analyses were not performed in this trial.

19) CONSORT: All important harms or unintended effects in each group

Not applicable.

19-i) Include privacy breaches, technical problems

Not applicable

19-ii) Include qualitative feedback from participants or observations from staff/researchers

Not applicable.

DISCUSSION

20) CONSORT: Trial limitations, addressing sources of potential bias, imprecision, multiplicity of analyses

20-i) Typical limitations in ehealth trials

"A limitation of the study is that the primary outcome, positive PANAS score, was self-reported. To respond accurately, participants must interpret the questions correctly, be aware of their emotional state and feelings and not be influenced by social desirability bias. In addition, as our trial recruited online, the control arm condition of prompting participants to use 'usual help-seeking strategies' may have meant that even control participants used online modalities to seek help which were encompassing of more conditions than Link, a similar issue to what we found in our pilot when we directed control arm participants to google."

21) CONSORT: Generalisability (external validity, applicability) of the trial findings

21-i) Generalizability to other populations

"The inclusion criteria were broad; however, our findings can only be generalised to young Australians aged 18-25 who use Facebook, Gumtree and/or Google to search for mental health related services or topics."

21-ii) Discuss if there were elements in the RCT that would be different in a routine application setting

"Work on young people accessing a web-based mental health support service in Australia (eheadspace) found that youth had high to very high levels of psychological distress but were at an earlier stage of illness than those presenting to their face to face service, which might explain our finding that young people using Link were less likely than the control arm to prefer formal sources of mental health care (Rickwood et al., 2016)."

22) CONSORT: Interpretation consistent with results, balancing benefits and harms, and considering other relevant evidence

22-i) Restate study questions and summarize the answers suggested by the data, starting with primary outcomes and process outcomes (use)

"This study tested whether Link, a website designed to guide young people to appropriate online and offline sources of mental health information and care, was effective in increasing psychological wellbeing and reducing barriers to seeking help for mental health problems among young people. Our results showed that Link did not increase positive affect immediately post intervention compared to usual online search strategies. Instead, we found that young people using Link and those using their usual search strategies both had a similar increase in positive affect of approximately 30% between baseline and three months."

22-ii) Highlight unanswered new questions, suggest future research

"Given the paucity of evidence for web-based help-seeking interventions the results of our trial and the Thought Spot trial will be important to compare in building up our understanding of mental health help-seeking interventions and the degree to which they are effective and efficient."

Other information

**23) CONSORT: Registration number and name of trial registry**
"The trial was registered with the Australian New Zealand Clinical Trials Registry on 20th November 2014 (Ref #: ACTRN126140012223628)."
**24) CONSORT: Where the full trial protocol can be accessed, if available**
Not applicable. The pilot feasibility study is cited in the manuscript.
**25) CONSORT: Sources of funding and other support (such as supply of drugs), role of funders**
"Funding was from the Young and Well Cooperative Research Centre, an Australian-based, international research centre (2011-2016) that united young people with researchers, practitioners, innovators, and policy-makers from over 70 partner organisations."
**X26-i) Comment on ethics committee approval**
"The study was approved by the University of Melbourne Human Research Ethics Committee (ID.1341063.4)."
**x26-ii) Outline informed consent procedures**
"Young people who met the eligibility criteria and provided informed consent registered for the trial using their email address and a self-generated password."
**X26-iii) Safety and security procedures**
"Email addresses and passwords allowed all participants to login and complete surveys at each wave and use the Link program (intervention arm only)."
**X27-i) State the relation of the study team towards the system being evaluated**
"It (Link) was designed by the research team in conjunction with young people and developed by the software company TigerSpike (https://tigerspike.com/)." and "The authors have no declared conflicts of interest."
